# Bayesian posterior density estimation reveals degeneracy in three-dimensional multiple emitter localization

**DOI:** 10.1038/s41598-023-49101-5

**Published:** 2023-12-16

**Authors:** Raymond van Dijk, Dylan Kalisvaart, Jelmer Cnossen, Carlas S. Smith

**Affiliations:** 1 Delft Center for Systems and Control, Delft University of Technology, Delft, 2628 CD The Netherlands; 2 Department of Imaging Physics, Delft University of Technology, Delft, 2628 CN The Netherlands

**Keywords:** Nanoscale biophysics, Techniques and instrumentation, Super-resolution microscopy

## Abstract

Single-molecule localization microscopy requires sparse activation of emitters to circumvent the diffraction limit. In densely labeled or thick samples, overlap of emitter images is inevitable. Single-molecule localization of these samples results in a biased parameter estimate with a wrong model of the number of emitters. On the other hand, multiple emitter fitting suffers from point spread function degeneracy, which increases model and parameter uncertainty. To better estimate the model, parameters and uncertainties, a three-dimensional Bayesian multiple emitter fitting algorithm was constructed using Reversible Jump Markov Chain Monte Carlo. It reconstructs the posterior density of both the model and the parameters, namely the three-dimensional position and photon intensity, of overlapping emitters. The ability of the algorithm to separate two emitters at varying distance was evaluated using an astigmatic point spread function. We found that for astigmatic imaging, the posterior distribution of the emitter positions is multimodal when emitters are within two times the in-focus standard deviation of the point spread function. This multimodality describes the ambiguity in position that astigmatism introduces in localization microscopy. Biplane imaging was also tested, proving capable of separating emitters up to 0.75 times the in-focus standard deviation of the point spread function while staying free of multimodality. The posteriors seen in astigmatic and biplane imaging demonstrate how the algorithm can identify point spread function degeneracy and evaluate imaging techniques for three-dimensional multiple-emitter fitting performance.

## Introduction

Single-molecule localization microscopy (SMLM)^1,2^ circumvents the diffraction limit through localization of sparsely activated emitters and reaches theoretical minimum uncertainty^3^. For localization, SMLM assumes a single molecule is contained in a region of interest (ROI). Overlap of emitter signal is inevitable in densely labeled samples and thick samples for 3D imaging. In SMLM, denser ROIs result in inaccurate estimates that have to be discarded.

Multiple emitter fitting^4–8^ mitigates this problem by extending the model to account for more than one emitter in the ROI. For two-dimensional localization, various high density localization methods exists, including temporal correlation^4^, compressed sensing^5,6^, deep learning^7^, and posterior density reconstruction^8^. These methods work by simultaneously estimating the model and parameters, or by making a model-free reconstruction. Fazel et al.^8^ used reversible jump Markov chain Monte Carlo (RJMCMC), a Bayesian method to sample directly from the posterior distribution, reconstructing the posterior by making a histogram of the samples. Bayesian approaches have the added advantages of including prior information and more accurately representing the uncertainty of model and parameter estimates. The RJMCMC sampler also makes model space jumps, changing the number of parameters while estimating the model.

**Figure 1:**
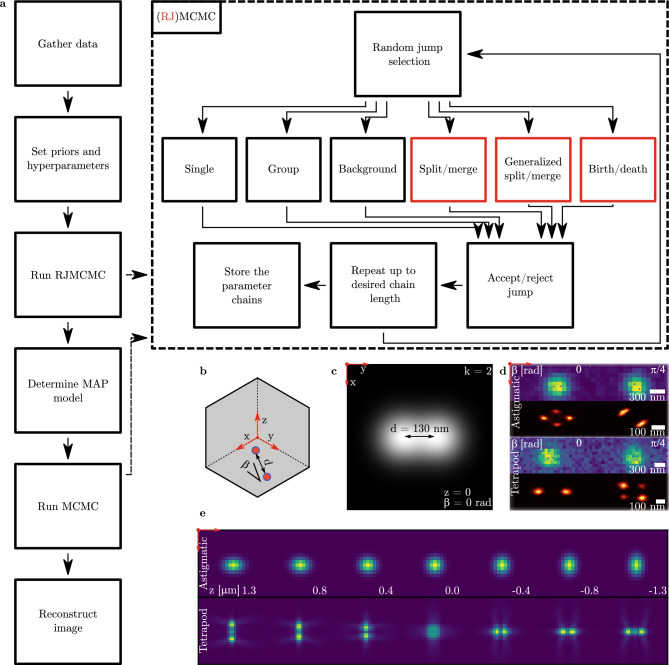
Schematic of the algorithm and problem description. **(a)** 3D RJMCMC localization flowchart. Frames are gathered and converted to photon counts. Then, priors and hyperparameters are set. An initial run of RJMCMC samples from the joint posterior of the parameters and model, from which the MAP number of emitters is determined. Using this MAP model, another MCMC run is used to condition the parameter distribution on the estimated model. Finally, the image is reconstructed by plotting histograms of the MCMC chains. The dashed rectangle demonstrates the (RJ)MCMC algorithm. Each loop, a move is randomly selected (RJMCMC moves that act on model space are highlighted in red) and used to propose a new set of parameters. This jump in parameters is accepted or rejected based on the ratio of posteriors. The algorithm repeats this loop, storing the parameters at each iteration to finally output the chain of iterations. **(b)** Diagram of two overlapping emitters separated by distance *d* and under angle $\beta$. **(c)** Ideal image of two nearby emitters. **(d)** Simulated data and reconstruction using astigmatic and tetrapod PSFs. Frames are shown in rows 1 and 3, while the reconstructed posterior distribution is shown in rows 2 and 4. **(e)** Z-scan of the PSFs used in d) to generate and reconstruct the data.

However, PSF degeneracy^9^ complicates multiple emitter fitting in three-dimensional localization. Figure 1b–d illustrates these problems with high density imaging and PSF degeneracy. As the PSF changes over depth, the image of an emitter at a given depth may match that of a sum of emitters at different depths. Figure 1e shows how an astigmatic and tetrapod PSF change over depth. This ambiguity increases model and parameter uncertainty, where a *k*-emitter model can be represented by a different number of emitters at different positions. It thus complicates the use of most 2D multiple emitter fitting methods for 3D, as they misrepresent these uncertainties.

In this article, we use Bayesian posterior density estimation to identify the PSF degeneracy in 3D multiple emitter fitting. To do so, we construct a 3D Bayesian localization algorithm using RJMCMC^10^. The algorithm is described in detail in the Supplementary Note and extends the approach from Fazel et al.^8^ to 3D. It provides an accurate reconstruction of the estimation uncertainty through posterior density sampling, constructing probability distributions for the number of emitters and their parameters. The reconstructed posteriors are used to quantify the circumstances in which 3D PSF degeneracy occurs in multiple emitter imaging.

Using this method, we show that multiple emitter fitting on two emitters with an astigmatic PSF results in degeneracy when the separation between the emitters is smaller than 2 standard deviations of the in-focus PSF ($\sigma _{\text {PSF}}$). Between $2\sigma _{\text {PSF}}$ and $1\sigma _{\text {PSF}}$, the multiple emitter fitting problem has two statistically equivalent solutions. Below $1\sigma _{\text {PSF}}$, the emitters are no longer individually identifiable. Additionally, we show that degeneracy in three-dimensional multiple emitter fitting can be avoided using biplane imaging.

Figure 1a shows a schematic of the algorithm. After gathering the frames and correcting for the camera gain, the user sets priors and hyperparameters that are appropriate for the imaging conditions. Then, an RJMCMC localization algorithm is ran on each of the frames, finding the posterior of the parameters and number of emitters given the data. Using the posterior, the maximum a posteriori (MAP) number of emitters is selected, and the estimates within this model are used to start a Markov chain Monte Carlo (MCMC) localization run. The MCMC output is used to form the histogram that reconstructs the object.

## Results

### Two emitter separability for astigmatic imaging

**Figure 2:**
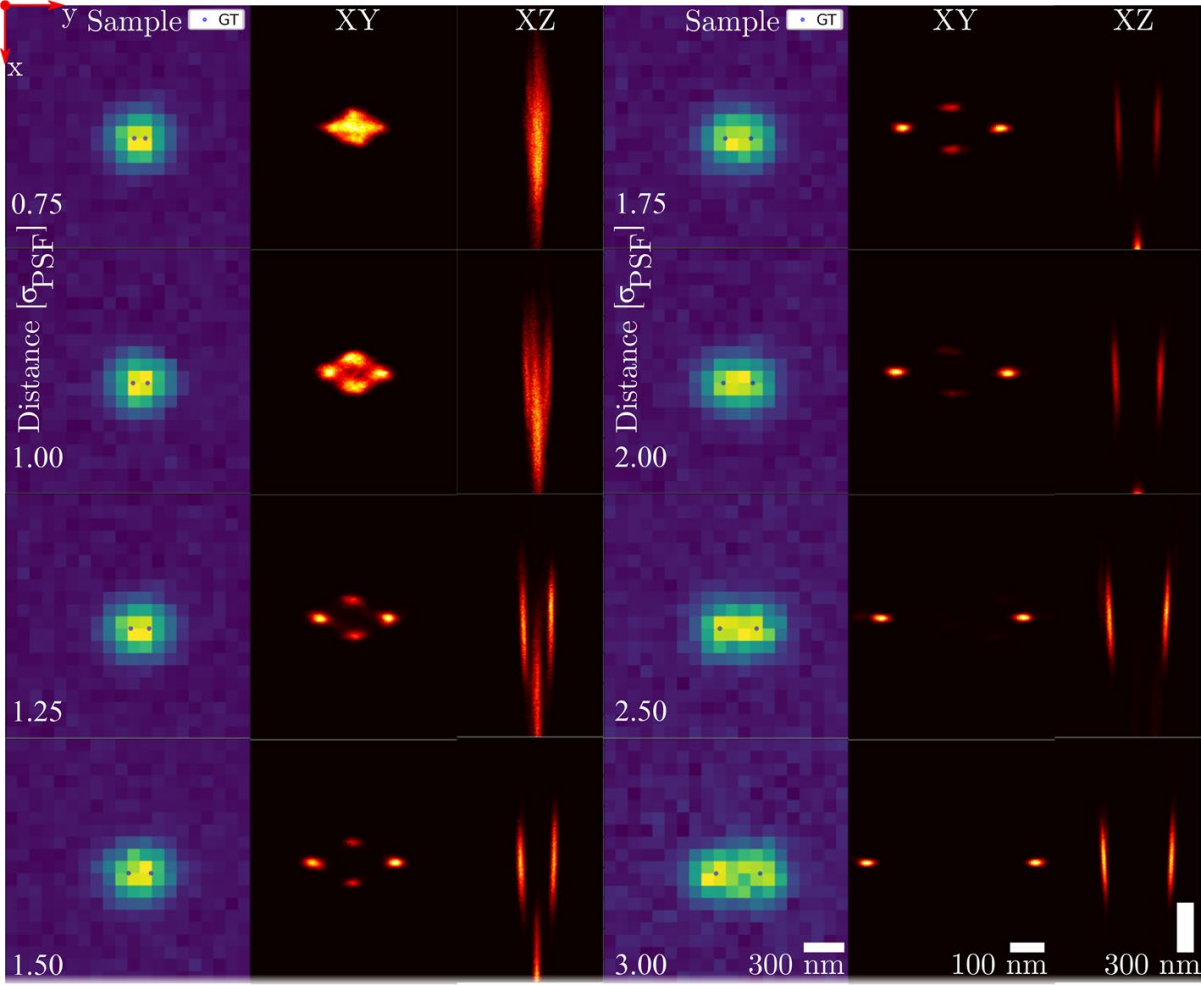
Two emitter separability using an astigmatic PSF, collecting 100 simulated frames into one reconstruction while varying emitter distance. *(Columns 1 and 4)* Example frames. *(Columns 2 and 5)* Zoomed in XY plane reconstruction. *(Columns 3 and 6)* Zoomed in XZ plane reconstruction. Emitters were placed in focal plane, with an intensity of 2000 photons each and a background of 20 photons. The ROI is 20 by 20 pixels, scalebars assume an effective pixel size of 100 nm. The reconstructed image consists of histograms from MCMC chains which used the MAP number of emitters as model.

We first evaluate the posterior distribution of multiple emitter fitting using an astigmatic PSF, as it is the most commonly used PSF for 3D localization. Two emitters were simulated at varying distance to one another, from 3$\sigma _{\text {PSF}}$ to 0.75$\sigma _{\text {PSF}}$, to analyze the ability to separate emitters. This is shown in Fig. 2. As MCMC generates samples from the posterior distribution, the reconstructions are made by plotting histograms of the MCMC chains for all of the 100 simulated frames. From Fig. 2, we see that the algorithm can separate the two emitters up to a distance of 2.5$\sigma _{\text {PSF}}$, as the histogram of the reconstructed posterior distribution shows two isolated peaks. For separations between 2$\sigma _{\text {PSF}}$ and 1$\sigma _{\text {PSF}}$, four peaks can be distinguished in the reconstructed posterior distribution, despite the MAP model finding two emitters. At a distance less than 1$\sigma _{\text {PSF}}$, the four individual peaks collapse into one cluster. Running a k-means clustering algorithm on the chain outputs for two clusters finds both clusters at the same position, in the middle of the frame. This shows the emitters can no longer be separated at distances lower than 1$\sigma _{\text {PSF}}$.

We further investigate the multimodal posterior distribution between 2$\sigma _{\text {PSF}}$ and 1$\sigma _{\text {PSF}}$. To investigate the multimodality, a single frame with four peaks in the reconstruction was analyzed as shown in Supplementary Fig. S12. The four peaks formed two pairs of possible modes, one at the true positions and one perpendicular to those. A chi-squared test was done to determine if either mode was representative of the frame. Interestingly, the chi-square values of 408 and 407 showed that both modes are representative for the frame, as the chi-square value at the 95% confidence interval is 456. We tested the probability of selecting the correct mode under both of these hypotheses. The probability of error was found to be 49.8%, making the modes indistinguishable in terms of likelihood. This confirms that the posterior distribution of the emitter position is indeed multimodal.

While the true mode localizations find the ground truth, the alternate mode localizations (at least for this particular astigmatic PSF model) are placed not just perpendicular to the ground truth, but also at a greater depth. At a separation of 0.75$\sigma _{\text {PSF}}$, the alternate mode is found at a depth of − 600 nm. This can be explained by the astigmatic PSF characteristics. When moving below the focal plane, the astigmatic PSF stretches along the same axis that separates the emitters. Thus, two emitters in the focal plane separated along the *x* axis can be represented by a pair on the *y* axis far below focal plane. This same problem occurs when emitters are separated along the *y* axis, resulting in an alternative pair along the *x* axis above the focal plane. These results show that posterior density reconstruction can be used to analyse 3D PSF suitability for high density localization microscopy. Specifically, these two special cases in which degeneracy occurs increase the maximum error that can be expected from 3D localization. This allows us to bound the worst-case localization error. Note that with randomly oriented emitters, only a subset of emitter pairs will be separated along a vector close to the *x* or *y* axis, so the impact on the mean squared error over all localizations will be limited.

From the *XZ* plots in Fig. 2, it can be seen that as the separation increases, the alternative mode starts to fall outside the provided PSF range of [− 1.3, 1.3] $\upmu$m, disappearing at large emitter separation. As the PSF range gets constrained further, this alternative mode disappears faster. This is consistent with the multimodality we found as a result of the PSF degeneracy, as constraining the PSF range decreases the solution space, thereby excluding ambiguous solutions.

### Influence of priors on multimodality

**Figure 3:**
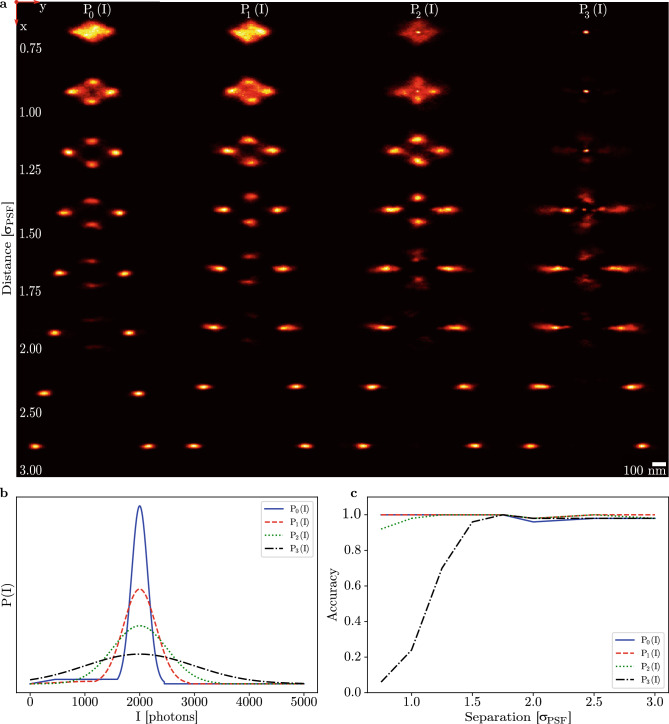
Two emitter separability using an astigmatic PSF, varying the width of the intensity prior, using the same frames as shown in Fig. 2. **(a)** XY plane reconstructions, each column using a different intensity prior. Images were formed by constructing histograms of the MCMC chains. **(b)** Plots of the respective intensity priors used in each column in **(a)**. **(c)** Accuracy of the found model as a function of the emitter separation plotted for each intensity prior used. Accuracy is found using $N_{\text {frames} ,\hat{k} = k} / N_{\text {frames}}$ with $N_{\text {frames} ,\hat{k} = k}$ the frames where the estimated model matches the ground truth and $N_{\text {frames} }$ the total frame count. The widths of the Gaussian peaks in the priors are 150, 300, 500, and 1000 photons, for $P_{0}(I)$ up to $P_{3}(I)$, respectively. $P_{0}(I)$ and $P_{1}(I)$ additionally use a uniform and sloped probability at lower intensities to facilitate model space moves. Priors were set to 0 at intensities beyond $2000 + 3 \sigma _{\text {prior}}$.

Fazel et al.^8^ identified that the intensity prior plays a critical role in multiple emitter fitting using RJMCMC. We therefore study the dependency of the multimodality on the intensity prior, as shown in Fig. 3. The image data from Fig. 2 was used for localization with four different intensity priors, $P_{0}(I)$ to $P_{3}(I)$. $P_{0}(I)$ and $P_{1}(I)$ combine a sloped and uniform probability at lower intensities with a Gaussian distribution around the expected count of 2000 photons, similar to what is done by Fazel et al.^8^. The intention of this prior is to increase the convergence speed of 3D RJMCMC by improving the ability to escape from local minima and to increase inter-model jumps. Priors $P_{2}(I)$ and $P_{3}(I)$ are strictly Gaussian. The standard deviation of the Gaussian peak in the intensity prior was varied from 150 up to 1000 photons, as shown in Fig. 3b. The first column of Fig. 3a matches the results of Fig. 2, as it uses the same priors and data. The algorithm separates emitters up to 2.5$\sigma _{\text {PSF}}$, multimodality is present from 2$\sigma _{\text {PSF}}$ down to 1$\sigma _{\text {PSF}}$, and emitters are no longer separable within 1$\sigma _{\text {PSF}}$. Using $P_{1}(I)$, column 2 again shows multimodality from 2$\sigma _{\text {PSF}}$ down to 1$\sigma _{\text {PSF}}$, with failure to separate emitters within 1$\sigma _{\text {PSF}}$. Using $P_{2}(I)$, multimodality is now observed at distances from 2$\sigma _{\text {PSF}}$ down to 1.25$\sigma _{\text {PSF}}$, failing to separate emitters within 1.25$\sigma _{\text {PSF}}$. Finally, using $P_{3}(I)$, multimodal reconstructions are found from 2$\sigma _{\text {PSF}}$ down to 1.5$\sigma _{\text {PSF}}$. Within this distance, the correct number of emitters is not found consistently. Figure 3c plots the accuracy of the estimated model for the priors used, calculated by counting the number of correct estimates and dividing by the frame count. For $P_{0}(I)$ and $P_{1}(I)$, the model accuracy is greater than 98% over the range of tested distances. For $P_{2}(I)$, model accuracy only goes below 95% at a distance of 0.75$\sigma _{\text {PSF}}$. Prior $P_{3}(I)$ decreases in model accuracy from a distance of 1.5$\sigma _{\text {PSF}}$, going from 94% down to 5% at a distance of 0.75$\sigma _{\text {PSF}}$. This shows that Gaussian intensity priors wider than 500 photons cannot consistently separate emitters within 1.5$\sigma _{\text {PSF}}$ of one another. While three out of four used priors can retrieve the model over 3$\sigma _{\text {PSF}}$ down to 0.75$\sigma _{\text {PSF}}$ distance, all of the used priors returned multimodal posterior densities within the range of 2$\sigma _{\text {PSF}}$ down to 1.5$\sigma _{\text {PSF}}$.

### Two emitter separability using biplane imaging

**Figure 4:**
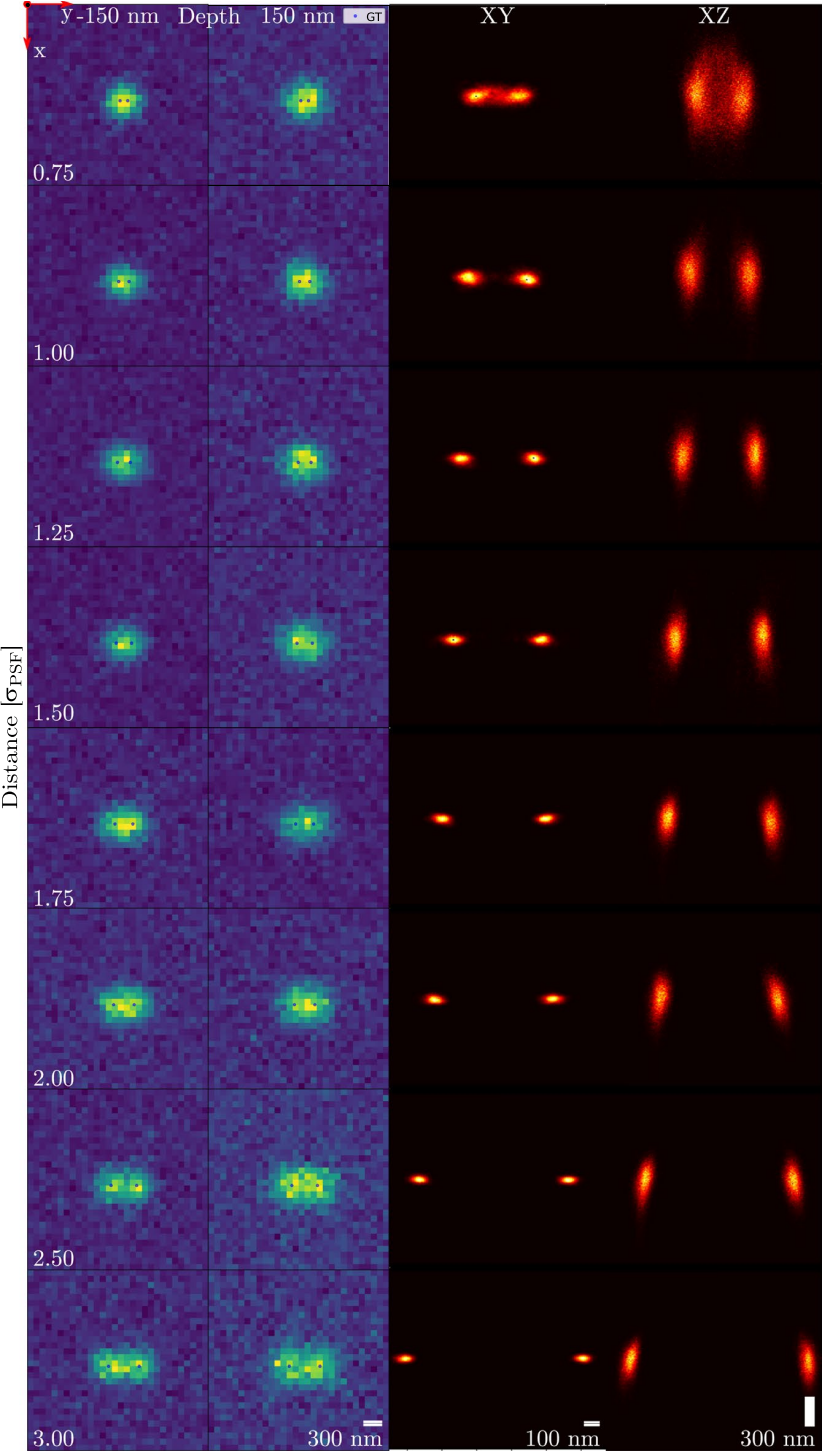
Two emitter separability using biplane imaging with planes separated by 300 nm. 100 pairs of frames were simulated and used to make one reconstruction. As photons are split evenly among the planes, the intensity prior was changed to be a Gaussian with mean 1000 photons and width 150 photons. *(Columns 1 and 2)* Example frames at both the positive and negative depth. *(Column 3)* XY plane reconstruction. *(Column 4)* XZ plane reconstruction. Emitters were placed in focal plane, with an intensity of 2000 photons each and a background of 20 photons. The ROI is 30 by 30 pixels, scalebars assume an effective pixel size of 100 nm. The reconstructed image consists of histograms from MCMC chains which used the MAP-estimated number of emitters.

Biplane imaging was tested for its ability to separate emitters and the multimodalities that may occur when doing so. For biplane imaging, the PSF can be approximated by a Gaussian. Unlike the astigmatic PSF, the 3D Gaussian PSF stays radially symmetrical over its range. It can therefore be expected that the same multimodality shown in astigmatic imaging will not be present here. Figure 4 shows that is indeed the case. As the emitter distance varies over the same range of 0.75$\sigma _{\text {PSF}}$ to 3$\sigma _{\text {PSF}}$, the algorithm consistently finds a model of two emitters while the reconstruction also consists of just two peaks. Under these conditions, biplane imaging can separate two emitters up to a distance of 0.75$\sigma _{\text {PSF}}$, entirely free of multimodality. This not only validates the idea that multimodality is caused by 3D PSF degeneracy, it also demonstrates how the algorithm can be used to determine which PSFs suffer the least from this problem and which are best used in dense 3D imaging.

## Conclusion

3D localization microscopy suffers from overlapping emitter images, often not being able to determine the number of active emitters in the ROI and leading to inaccurate position estimates. Multiple emitter fitting algorithms can find the number of emitters, but the added complexity of 3D PSF degeneracy means that these algorithms often misrepresent the uncertainty of their estimates.

We constructed 3D RJMCMC to identify PSF degeneracy in multi-emitter fitting problems, by using the reconstructed posterior density of emitter positions. For astigmatic and biplane imaging, 3D RJMCMC is capable of separating emitters up to a distance of 1$\sigma _{\text {PSF}}$ and 0.75$\sigma _{\text {PSF}}$, respectively, localizing emitters in 3D where SMLM methods would fail. However, astigmatic imaging at these densities will result in multimodal reconstructions. This is an accurate representation of the posterior and a consequence of the 3D PSF structure. Therefore, posterior density reconstruction is the tool of choice to identify potential PSF degeneracy problems in dense 3D localization.

## Discussion

As a main result of our 3D RJMCMC analysis, we found that single-frame astigmatic multiple emitter fitting can result in a multimodal posterior distribution. Due to the practicality of astigmatic imaging and its ability to acquire high-density data, this should serve as an important warning when using it for multiple emitter localization.

Fortunately, the multimodality in multiple emitter fitting using astigmatic imaging can be reduced by analyzing a larger time window or by limiting the PSF depth range. Constraining the range reduces the variety of shapes the PSF can take on, limiting PSF degeneracy. Multimodality may also be reduced by encoding the axial position in intensity. Modifying the algorithm to work with total internal reflection fluorescence (TIRF)^11,12^ is therefore a promising method to image without multimodality. Modulation-enhanced localization microscopy (meLM)^13^ techniques such as ModLoc^14^, SIMFLUX^15^, and ROSE^16^ could all be decoded with 3D RJMCMC localization and a position-dependent intensity prior. A combination of these techniques with RJMCMC may result in a posterior distribution free of multimodality.

With our analysis, we show the importance of including the localization uncertainty in localization algorithms. In multiple emitter fitting, 3D RJMCMC reveals the multimodality of the posterior. This allows us to reveal degeneracy, whereas traditional point estimates would have resulted in overconfident position estimates. Furthermore, 3D RJMCMC also shows that the position uncertainty for individual emitters is not well-represented by Gaussian uncertainty ellipses with a diagonal covariance matrix. Future research should therefore look at incorporating the full uncertainty covariance matrix into the localization algorithm.

The 3D RJMCMC algorithm is best used to analyze imaging techniques for their effectiveness in 3D multiple emitter fitting. Though no multimodality was revealed when testing biplane imaging with 3D RJMCMC localization, orientations of emitter pairs were not exhaustively tested. Furthermore, 3D RJMCMC assumes the PSF model to be accurately known during localization. In the case of PSF uncertainty, we expect this results in an increase of the localization uncertainty contained in the posterior. Within the current methodological framework of 3D RJMCMC, there is however no obvious way to incorporate a PSF mismatch. This combined with the slow and memory intensive nature of RJMCMC means we do not recommend using the algorithm for localization.

However, it should be used to analyze PSFs for possible multimodality in dense samples. Specifically, it remains an open question which PSF allows for both the evasion of degeneracy and an accessible implementation. Additionally, we recommend studying the occurrence of multimodality of the tetrapod PSF, as it is a popular choice for 3D imaging. Our initial study (see Supplementary Note) suggests that multimodality occurs for multiple emitter localization with the tetrapod PSF at 45-degree angles with respect to the *x* axis. Further study is needed into the conditions, such as the emitter separation, in which this multimodality occurs. 3D RJMCMC is the method of choice to study this. Testing imaging techniques that would require a position dependent intensity prior, such as TIRF or meLM techniques, is also a topic of great interest.

## Methods

### Image formation model and key probabilities

For multiple emitter fitting, the expected photon count per camera pixel can be described as:

1 $$\begin{aligned} \mu _{i} = \sum _{j = 1}^{k} \theta _{I,j} \int _{A_k} H(\theta _{x,j},\theta _{y,j},\theta _{z,j}) \text {d}x \text {d}y + \theta _{b}, \end{aligned}$$

with $\mu _{i}$ the photon count in pixel *i*, $\theta _{I,j}$ the intensity of the $j{\text {th}}$ emitter in the frame, *k* the amount of emitters in the frame, $A_k$ the pixel area, *H*(*x*, *y*, *z*) the PSF, $\theta _{x,j}$, $\theta _{y,j}$, $\theta _{z,j}$ the 3D position of the *j*th emitter, and $\theta _{b}$ the background photon count. For a high gain camera, such as an electron multiplying charge coupled device (EMCCD), the readout noise is negligible and thus the likelihood function has a Poisson distribution:

2 $$\begin{aligned} P(D_i|\theta , k) = \frac{\mu _{i}^{D_{i}}\exp \left( -\mu _{i}\right) }{D_{i}!}, \end{aligned}$$

with $P(D_i|\theta , k)$ the likelihood of observing measured data *D* on the $i{\text {th}}$ pixel as function of parameter vector $$\theta = \begin{bmatrix} \theta _{x,0}&\theta _{y,0}&\theta _{z,0}&\theta _{I,0}&\dots&\theta _{x,k}&\theta _{y,k}&\theta _{z,k}&\theta _{I,k}&\theta _{b} \end{bmatrix}$$ and number of emitters *k*. Given the pixels are independent, the likelihood of one frame becomes:

3 $$\begin{aligned} P(D|\theta , k) = \prod _{i=1}^{N_{\text{p} }} \frac{\mu _{i}^{D_{i}}\exp \left( -\mu _{i}\right) }{D_{i}!}, \end{aligned}$$

with $N_{\text{p} }$ the pixel count. The joint posterior distribution of the parameters and model can then be found using Bayes’ rule:

4 $$\begin{aligned} P(\theta , k | D) = \frac{P(D | \theta , k) P(\theta | k) P(k)}{P(D)}, \end{aligned}$$

with *P*(*k*) the model prior, $P(\theta |k)$ the parameter prior given the model, and *P*(*D*) the evidence. The priors can be formulated from earlier attained knowledge of labeling density or emitter intensity, however the evidence term, $P(D) = \int P(D | \theta , k) P(\theta | k) P(k) \text {d} \theta \text {d} k$, only has a closed form solution when the prior distribution is conjugate to the posterior. This is often not the case^17^, therefore Reversible jump Markov chain Monte Carlo (RJMCMC)^10^ is employed to asymptotically sample from the posterior.

After attaining samples from $P(\theta , k | D)$, the maximum a posteriori (MAP) number of emitters, $\hat{k}$, is used as the true model to condition the parameter estimate on, running a Markov chain Monte Carlo (MCMC) algorithm to find $P(\theta | \hat{k}, D)$. This is done to avoid introducing bias in the parameter estimates coming from models of different dimension.

### Priors and hyperparameters

The algorithm takes in priors for the 3D position, emitter intensity, background intensity, and emitter count. Throughout the tests, the prior on the lateral emitter position is kept uniform over the ROI plus four extra pixels, to account for the influence of emitters outside the ROI. The axial position prior is uniform over the presumed depth range of the PSF. Emitter intensity was set as a Gaussian distribution, enabling the algorithm to separate the emitters. In practice, it is recommended^8^ to estimate the intensity prior using kernel density estimation on intensity data of a previous SMLM run. Finally, the emitter count prior is also kept uniform. Although it is possible estimate the emitter count within a sample given the label density, it can still vary widely on a local scale, thus we keep the prior uniform for smaller ROIs.

Each iteration, the move was randomly selected using user-determined selection probabilities [$P_{\text{single} }$, $P_{\text{group} }$, $P_{\text{background} }$, $P_{\text{split} }$, $P_{\text{merge} }$, $P_{\text{g-split} }$, $P_{\text{g-merge} }$, $P_{birth }$, $P_{\text{death} }$]. Throughout the tests, the RJMCMC burn-in portion uses [1/5, 1/5, 1/5, 1/15, 1/15, 1/15, 1/15, 1/15, 1/15], while post burn-in [1/4, 1/4, 1/4, 0, 0, 3/32, 3/32, 1/32, 1/32] are used. The burn-in portion uses higher model space move probabilities to ensure more model space mixing, while post burn-in focuses more on parameter space moves. The MCMC portion uses [2/5, 2/5, 1/5, 0, 0, 0, 0, 0, 0], focusing mainly on emitter parameters. Each test runs for 30,000 RJMCMC iterations, using 10,000 of those as burn-in and following them up with 5000 MCMC iterations. The parameter space moves use random walk samplers, leaving jump sizes [$\sigma _{x}$, $\sigma _{y}$, $\sigma _{z}$, $\sigma _{I}$, $\sigma _{b}$] as parameters for tuning. For good mixing, the lateral jump size may vary from 0.05 to 0.1 pixels, the axial jump size from 0.07 up to 0.12 $\upmu$m, the emitter intensity between 10 and 40 photons, and the intensity between 1 to 3 photons.

### Convergence and precision

To verify convergence of the algorithm, 100 frames with a single active emitter were localized. Emitters in the center of the ROI sampled their intensity randomly from the matching prior and were given a random sub-pixel displacement. Supplementary Figures S3 and S5 show the model and parameter autocorrelation as well as the time series and histogram of the model, for a high and low signal to background, respectively. The algorithm manages to converge to the correct model 100% of the time, while also converging in parameter space. By using only uniform priors, the algorithm yields an unbiased estimate that can be compared to the Cramér–Rao lower bound (CRLB)^3^ to verify the precision. For non-uniform priors, the Van Trees inequality (VTI)^18,19^ can be used as a Bayesian Cramér–Rao bound to find the theoretically minimum localization error. Supplementary Figure S8 shows a violin plot of the precisions found with 3D RJMCMC localization compared to the CRLB over varying emitter intensity. It can be seen that the localization precision matches the CRLB over the plotted intensity range. Supplementary Figure S9 shows violin plots for the root mean squared error of the same data. The results show that the algorithm reaches the minimum theoretical uncertainty for low emitter density.

### Synthetic data and results

For Fig. 2, two emitters were placed in focus and simulated using an astigmatic PSF, their center of mass in the middle of the ROI. The PSF was evaluated using a 3D Gaussian approximation^3^, with parameters [$s_{0,x}$, $\gamma _{x}$, $d_{x}$, $A_{x}$] and [$s_{0,y}$, $\gamma _{y}$, $d_{y}$, $A_{y}$] of [$\sigma _{\text {PSF} }$, 2, 3, 0] and [$\sigma _{\text {PSF}}$, -2, 3, 0], respectively. Here $\sigma _{\text {PSF}}$ is the width in focal plane, set at 1.2 pixels. The PSF range was set at [− 1.3, 1.3] $\upmu$m, with a ROI size of 20 by 20 pixels. Emitter intensity was fixed at 2000 photons, with a background intensity of 20 photons. A total of 100 frames were simulated and their 5000 iteration MCMC chains were merged to finally form the histogram reconstructions of the *XY* and *XZ* planes. The histograms were magnified in *x* and *y* direction by a factor 2.5 with respect to the sample frames. Move selection probabilities were as in Subsection Priors and hyperparameters, while the jump size hyperparameters were set to [0.1, 0.1, 0.08, 15, 1]. All priors were kept uniform except the emitter intensity, using a Gaussian around 2000 photons with a width of 150 and a small uniform probability between 0 and 1500 that slopes down to 0, as shown in Fig. 3b. The prior is set to 0 for intensities greater than 2450 photons. The number of emitters ranges from 0 to 6 and lateral position estimates may exceed the ROI by four pixels. Background intensity was limited to a range of 1 to 40 photons.

Figure 3 uses the same data and settings as Fig. 2, only changing the random number generator seed and the emitter intensity priors used. Priors $P_{0}(I)$ to $P_{3}(I)$ all use Gaussian distributions centered around 2000 photons, with a width of 150, 300, 500, and 1000 photons, respectively. Again, $P_{0}(I)$ and $P_{1}(I)$ keep a uniform probability sloping to 0 at lower intensities to help facilitate splitting of emitters.

Figure 4 uses 100 frames simulated with biplane imaging, splitting the response of a Gaussian PSF between planes at + 150 and − 150 nm depth relative to the focal plane. A depth range of [− 1, 1] $\upmu$m was used. Again, the PSF was evaluated with a 3D Gaussian approximation^3^ of an experimentally measured astigmatic PSF on the setup described in Ref.^20^, with [1.70, $-4.64$, 8.34, 0.00] for the *x* and *y* parameters. Emitter intensities and background photons were split evenly across the planes. The ROI is now expanded to 30 by 30 pixels. All hyperparameters and priors used were identical to the previous experiments, except the emitter intensity prior, which is a Gaussian with mean 1000 and width 150 photons. The intensity prior is set to 0 for intensities greater than 1450 photons and again is uniform between 200 and 500 photons, sloping upwards from 0 to 200 photons.

### Supplementary Information

Supplementary Information.

## Data Availability

The data and code are available online from the Github repository at https://github.com/qnano/rjmcmc3D. Two Jupyter notebook examples are also included and can be run with Google colaboratory at https://colab.research.google.com/github/qnano/rjmcmc3D/blob/master/colab_example.ipynb and https://colab.research.google.com/github/qnano/rjmcmc3D/blob/master/colab_example_cspline.ipynb.
