# Supplementary material for: Bayesian posterior density estimation reveals degeneracy in three-dimensional multiple emitter localization
Source: Sci Rep. 2023 Dec 16;13:22372. doi: 10.1038/s41598-023-49101-5 (PMC10724183; doi:10.1038/s41598-023-49101-5)
Supplement: Supplementary file 1 — Supplementary Information. [file 41598_2023_49101_MOESM1_ESM.pdf]

Supporting information for: Bayesian posterior density estimation reveals degeneracy in three-dimensional multiple emitter localization

Raymond van Dijk[1,+], Dylan Kalisvaart[1,+], Jelmer Cnossen[1], and Carlas S. Smith[1,2,*]

[1]Delft Center for Systems and Control, Delft University of Technology, Delft, the Netherlands
[2]Department of Imaging Physics, Delft University of Technology, Delft, the Netherlands
[*]c.s.smith@tudelft.nl
[+]these authors contributed equally to this work

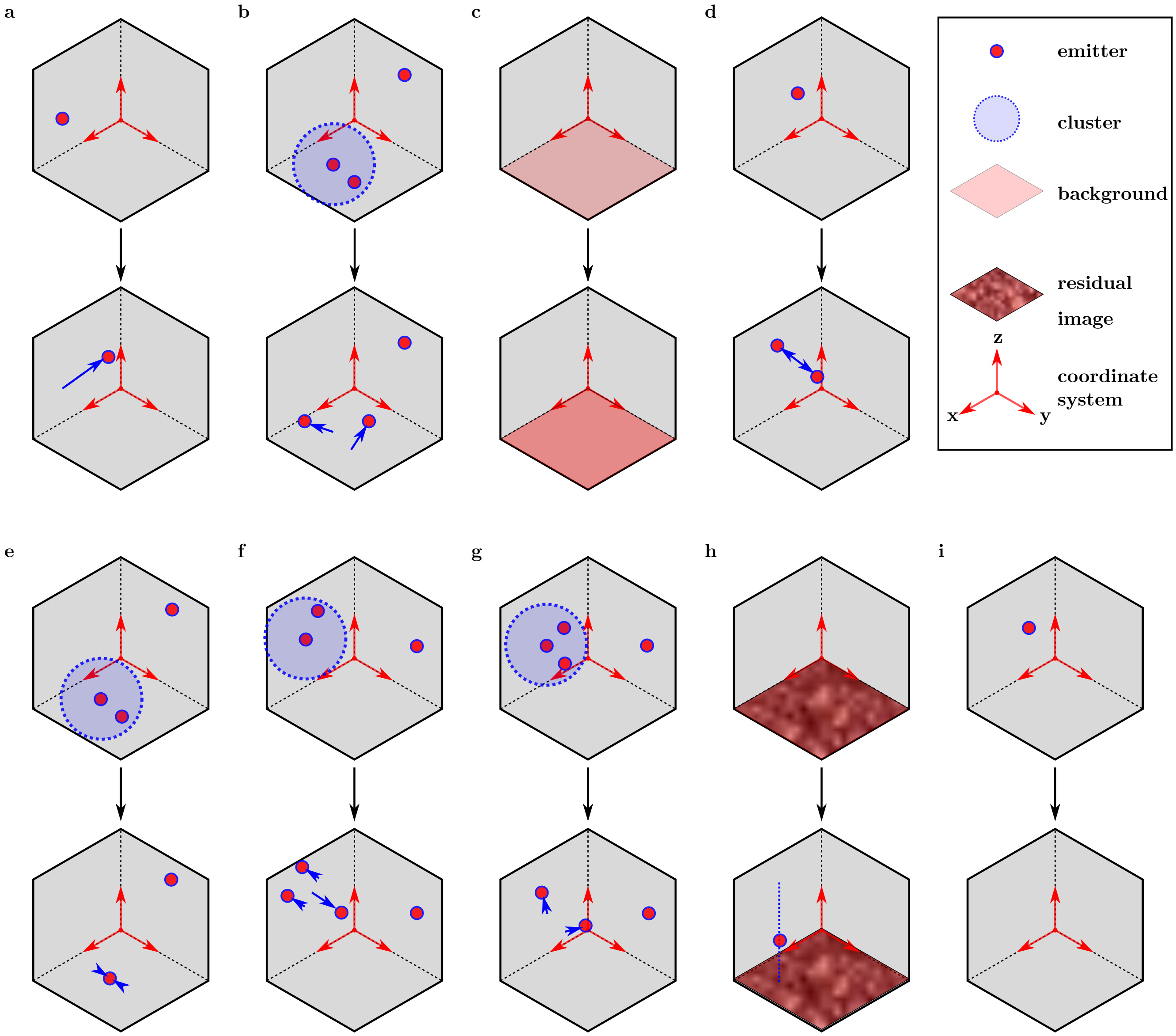

Supplementary Figure S1. A collection of diagrams visualising the proposals that each move makes. **a)** The single emitter move changes the 3D position of an emitter, as well as its intensity. **b)** The group move finds a cluster of emitters and randomly and independently changes their positions and intensities. **c)** The background move changes the estimated background photon count. **d)** Split distributes the intensity of a random emitter over two new emitters. **e)** Merge randomly selects an emitter and searches within a given radius for an emitter to merge it with, combining intensities. **f)** Generalized split finds a cluster of N emitters and splits them off into $N+1$ emitters. **g)** Generalized merge finds a cluster of $N+1$ emitters to merge into N emitters. **h)** Birth constructs a residual image to use as a probability distribution for possible undetected emitter positions, generating a new emitter with a random position. **i)** Death removes a randomly selected emitter from the model.

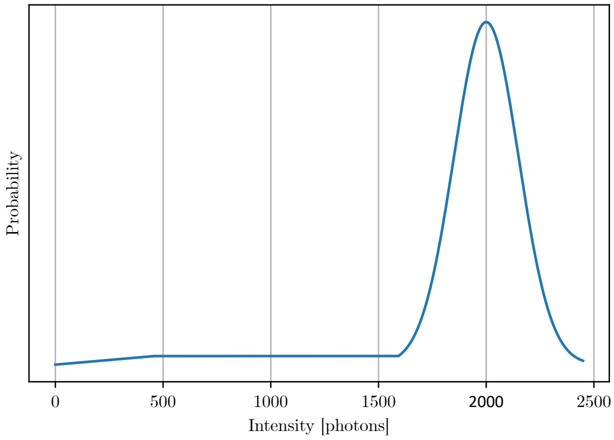

Supplementary Figure S2. Prior on intensity. Used for synthetic data with intensities drawn from $\mathcal{N}(2000, 150)$.

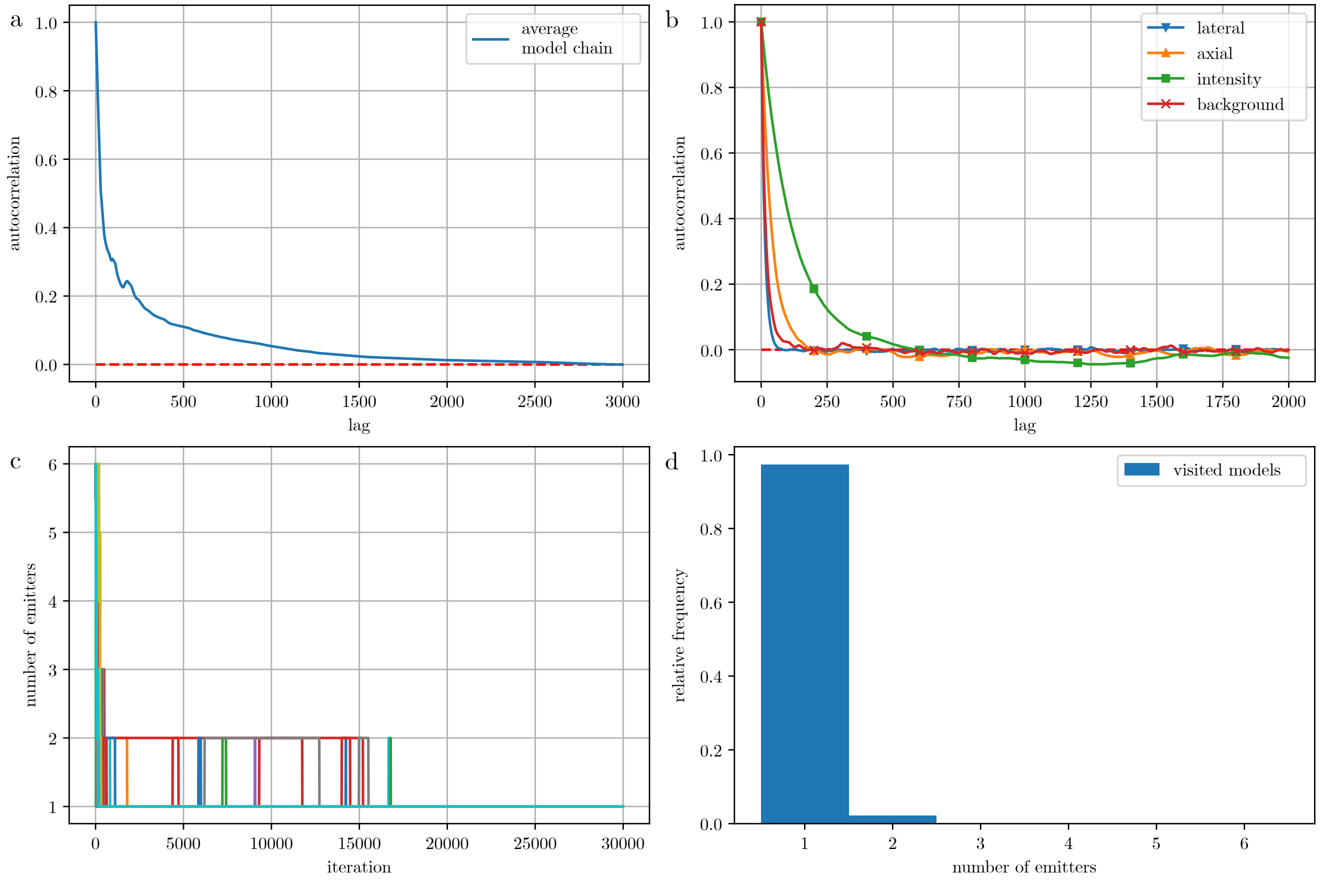

Supplementary Figure S3. Model and parameter space convergence of the algorithm for high SBR frames. **a)** Autocorrelation of the model chain. **b)** Autocorrelation of the parameters. **c)** Time series plot of the model for all frames. **d)** Histogram of all visited models across all frames. Emitter intensities were sampled from $\mathcal{N}(2000, 150)$, background was set to 20 photons. Emitters were placed in focal plane at the center of the region of interest (ROI) and given a sub-pixel shift drawn from $\mathcal{U}(-0.5, 0.5)$. 100 frames were used.

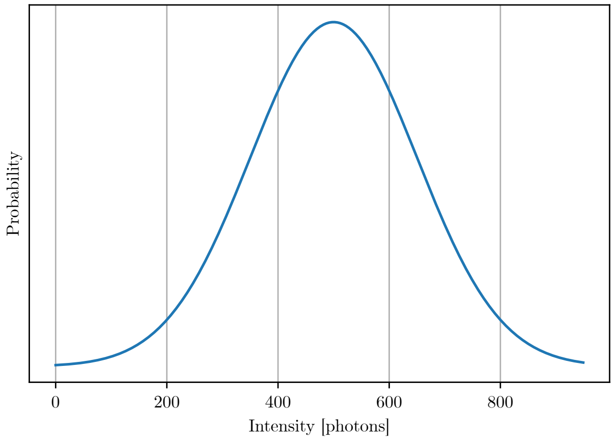

Supplementary Figure S4. Prior on intensity for low SBR. Used for synthetic data with intensities drawn from $\mathcal{N}(500,150)$.

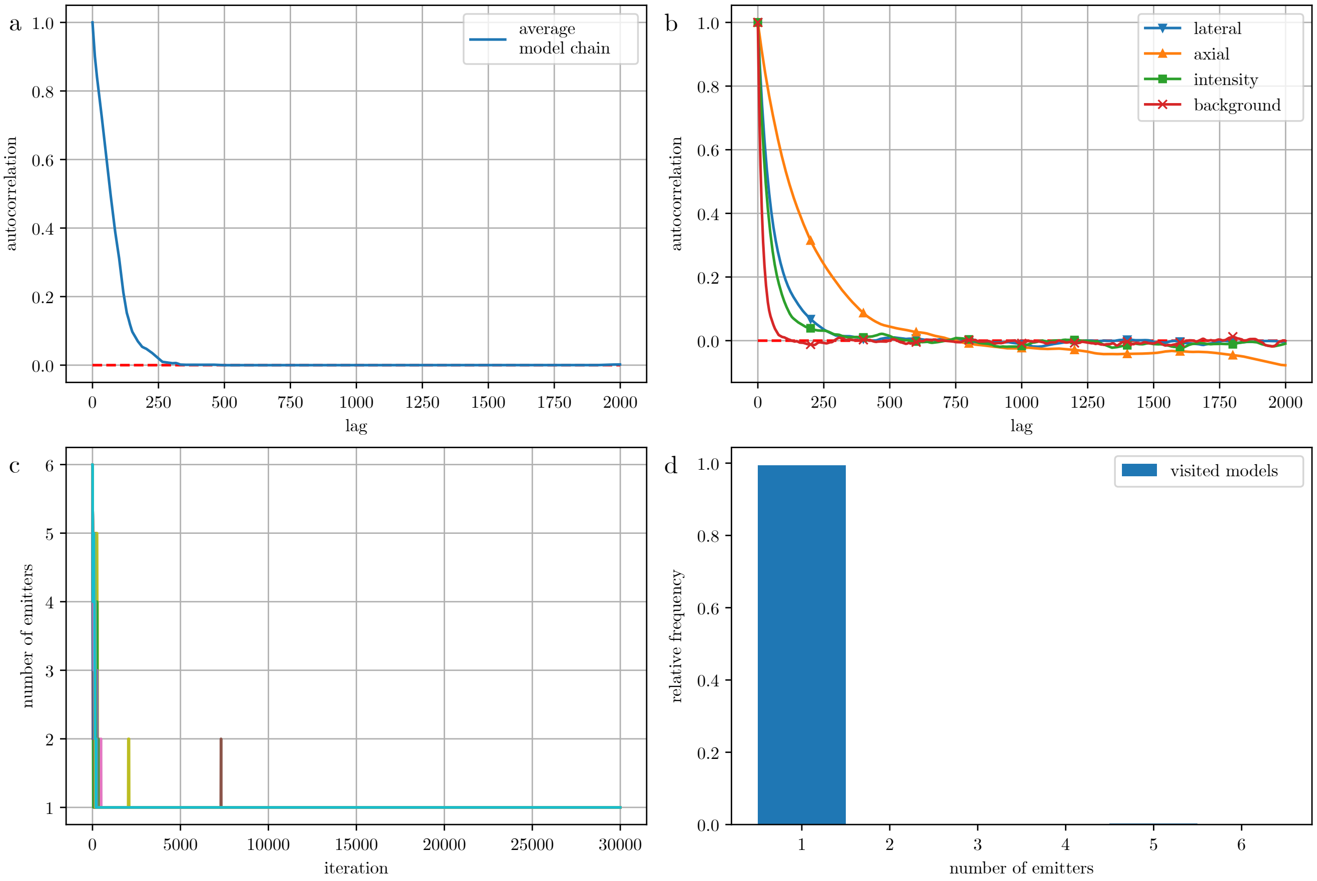

Supplementary Figure S5. Model and parameter space convergence of the algorithm for low SBR frames. **a)** Autocorrelation of the model chain. **b)** Autocorrelation of the parameters. **c)** Time series plot of the model for all frames. **d)** Histogram of all visited models across all frames. Emitter intensities were sampled from $\mathcal{N}(500,150)$, background was set to 20 photons. Emitters were placed in focal plane at the center of the ROI and given a sub-pixel shift drawn from $\mathcal{U}(-0.5,0.5)$. 100 frames were used.

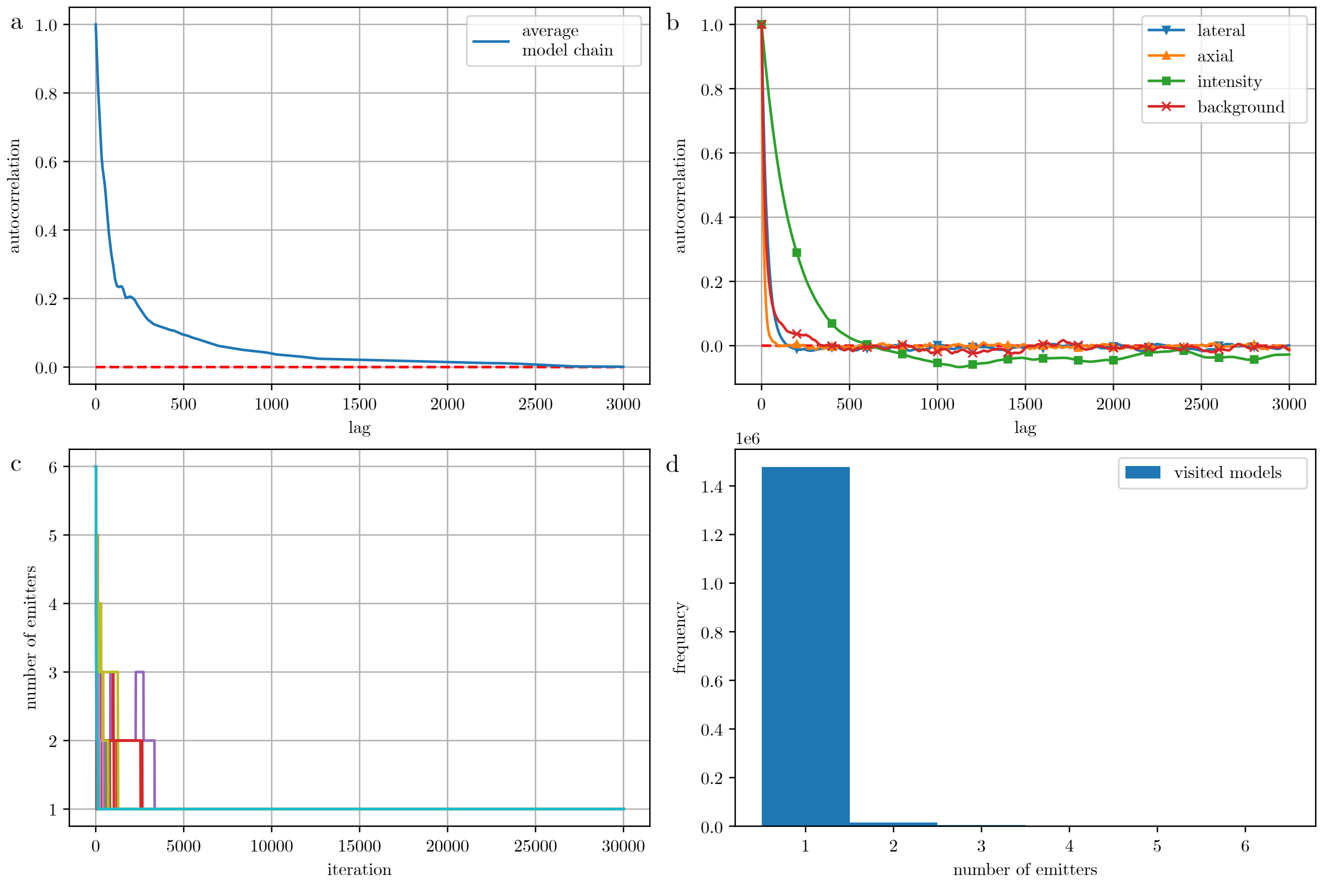

Supplementary Figure S6. Model and parameter space convergence of the algorithm for high SBR frames. **a)** Autocorrelation of the model chain. **b)** Autocorrelation of the parameters. **c)** Time series plot of the model for all frames. **d)** Histogram of all visited models across all frames. Emitter intensities were sampled from $\mathscr{N}(2000, 150)$, background was set to 20 photons. Emitters were placed in focal plane at the center of the ROI and given a sub-pixel shift drawn from $\mathscr{U}(-0.5, 0.5)$. 100 frames were used.

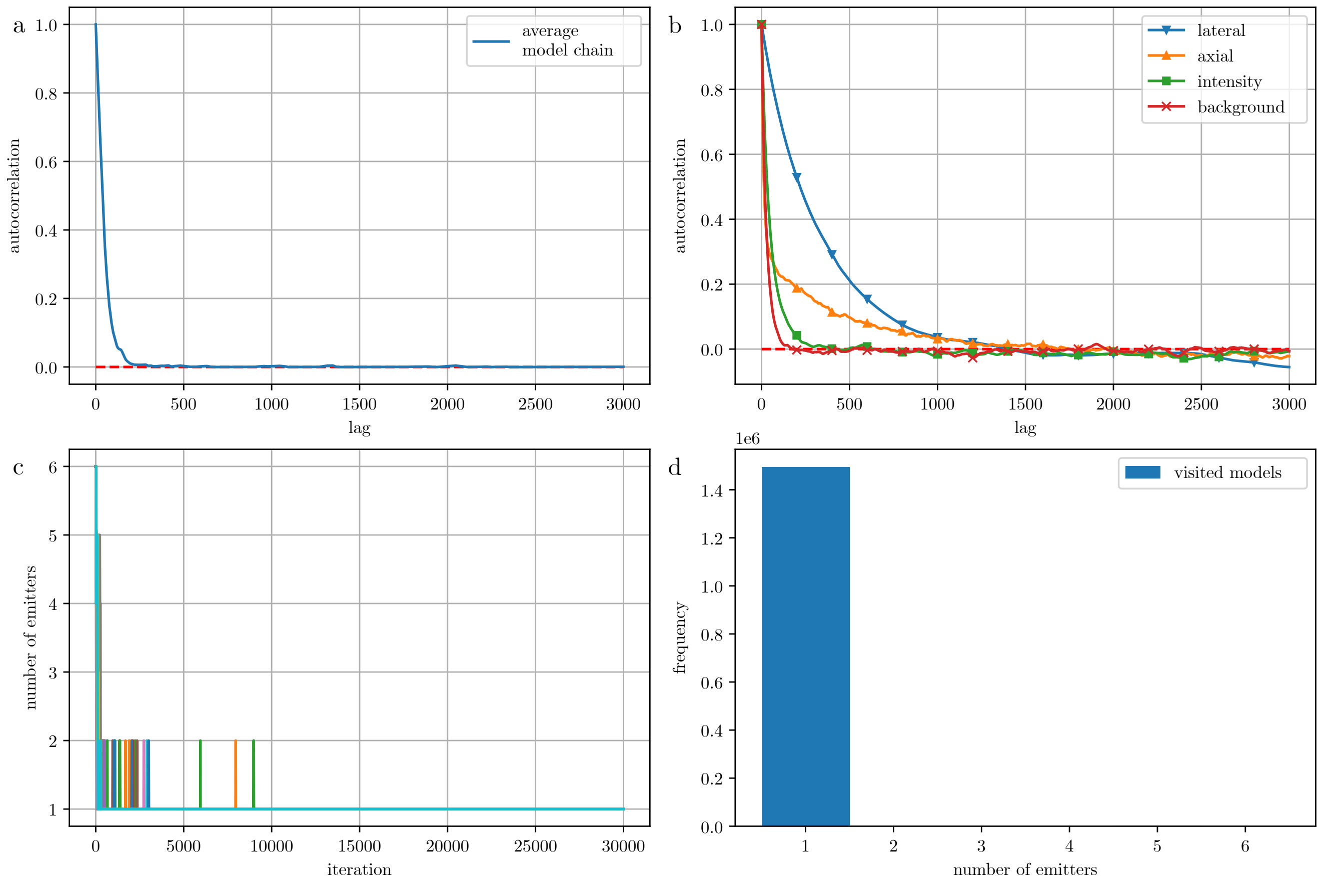

Supplementary Figure S7. Model and parameter space convergence of the algorithm for low SBR frames. **a)** Autocorrelation of the model chain. **b)** Autocorrelation of the parameters. **c)** Time series plot of the model for all frames. **d)** Histogram of all visited models across all frames. Emitter intensities were sampled from $\mathscr{N}(500, 150)$, background was set to 20 photons. Emitters were placed in focal plane at the center of the ROI and given a sub-pixel shift drawn from $\mathscr{U}(-0.5, 0.5)$. 100 frames were used.

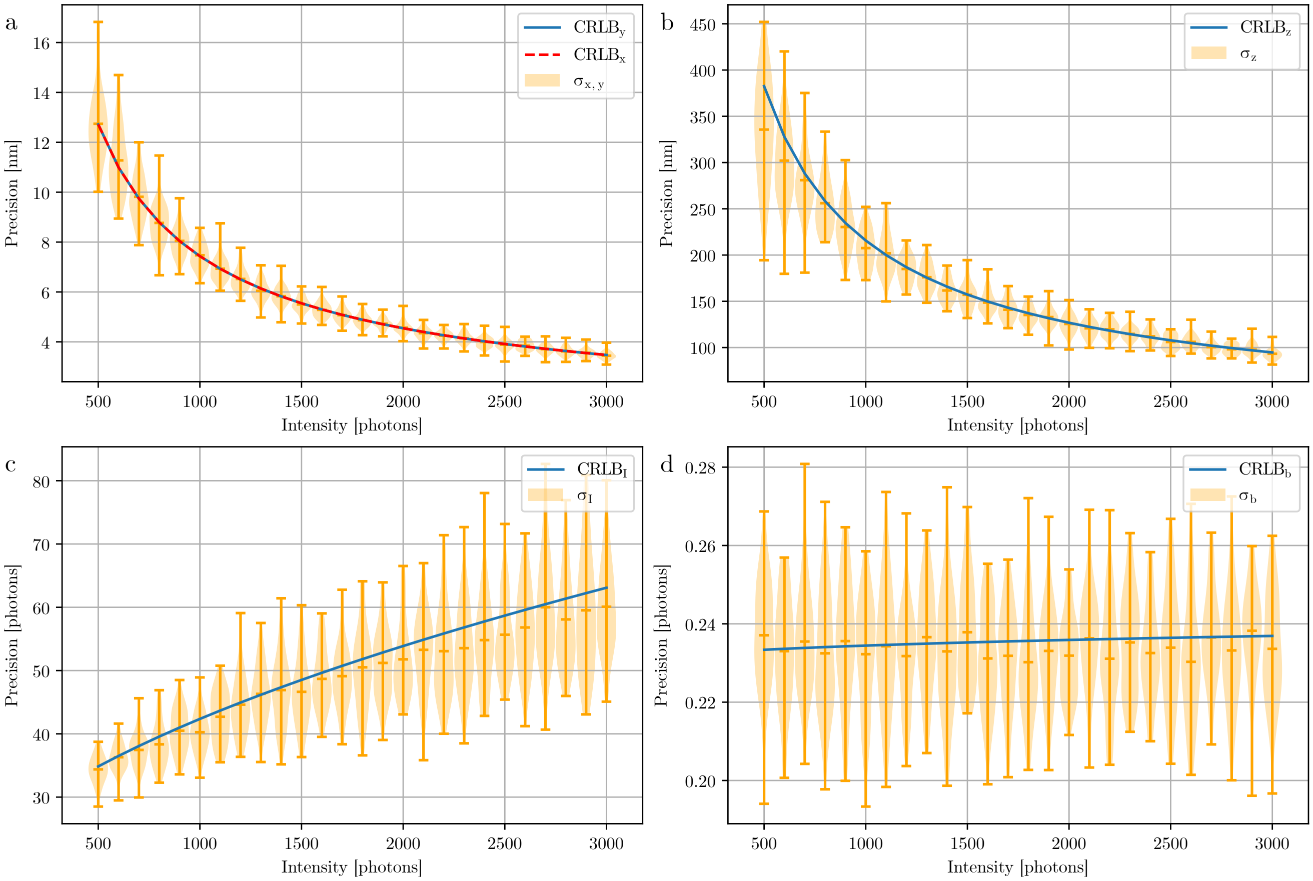

Supplementary Figure S8. Violinplots of parameter estimates over intensity using astigmatic imaging, compared to the CRLB. **a)** Lateral precision violinplot, using an effective pixel size of 100 nm. **b)** Axial precision violinplot. **c)** Violinplot of the emitter intensity precision. **d)** Violinplot of the background intensity precision. Precisions were calculated from the MCMC chain of individual frames, assuming a Gaussian distribution. With a background of 20 photons, 50 frames were used per datapoint from 500 to 3000 emitter intensity photons. Emitters were placed in focal plane at the center of the ROI and given a sub-pixel shift drawn from $\mathscr{U}(-0.5, 0.5)$.

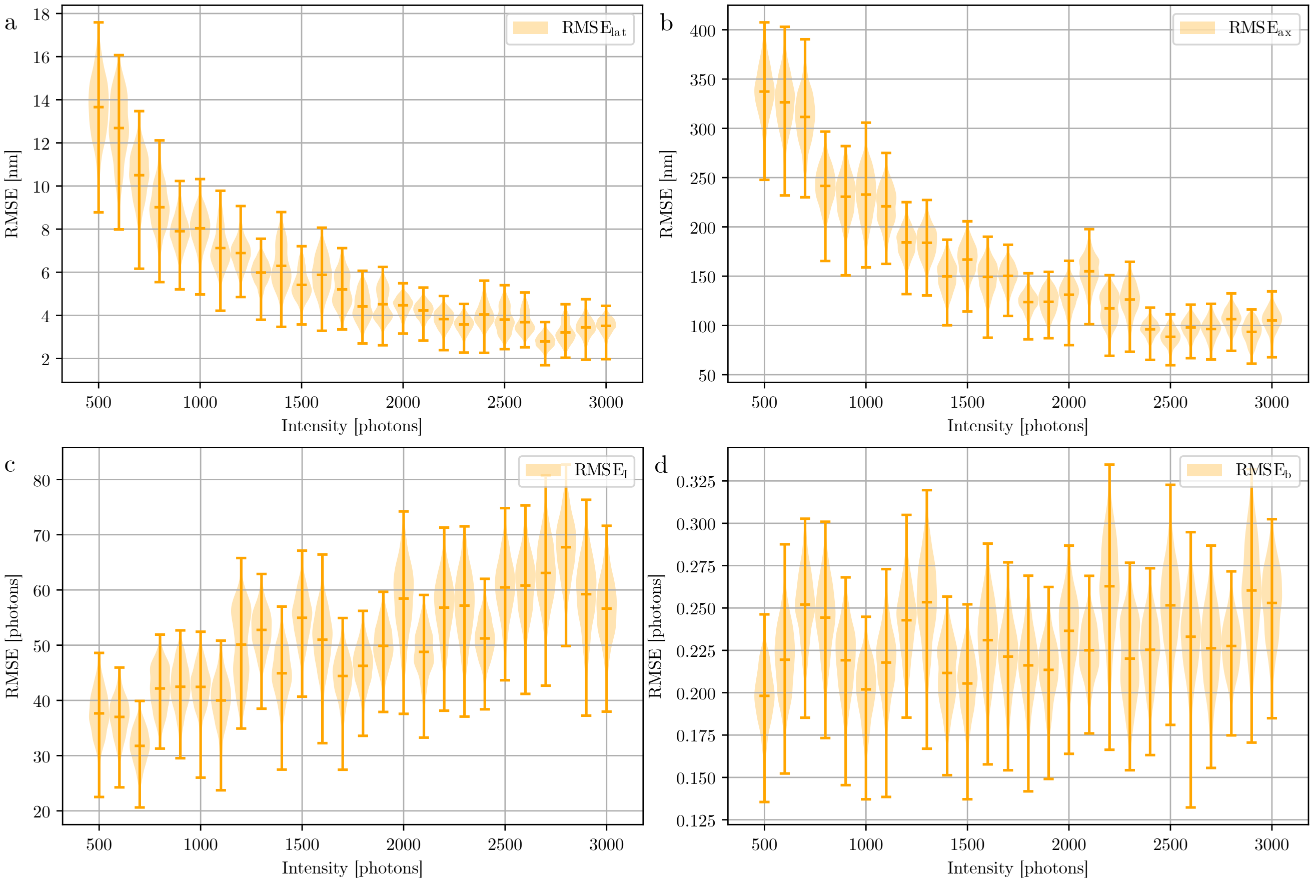

Supplementary Figure S9. Bootstrap resampled root mean square error (RMSE) results for the parameter estimates plotted over intensity using astigmatic imaging. **a)** Violinplot of the lateral RMSE. **b)** Violinplot of the axial RMSE. **c)** Violinplot of the emitter intensity RMSE. **d)** Violinplot of the background intensity RMSE. Results were resampled using 50% of the available data and 1000 runs. With a background of 20 photons, 50 frames were used per datapoint from 500 to 3000 emitter intensity photons. Emitters were placed in focal plane at the center of the ROI and given a sub-pixel shift drawn from $\mathscr{U}(-0.5, 0.5)$.

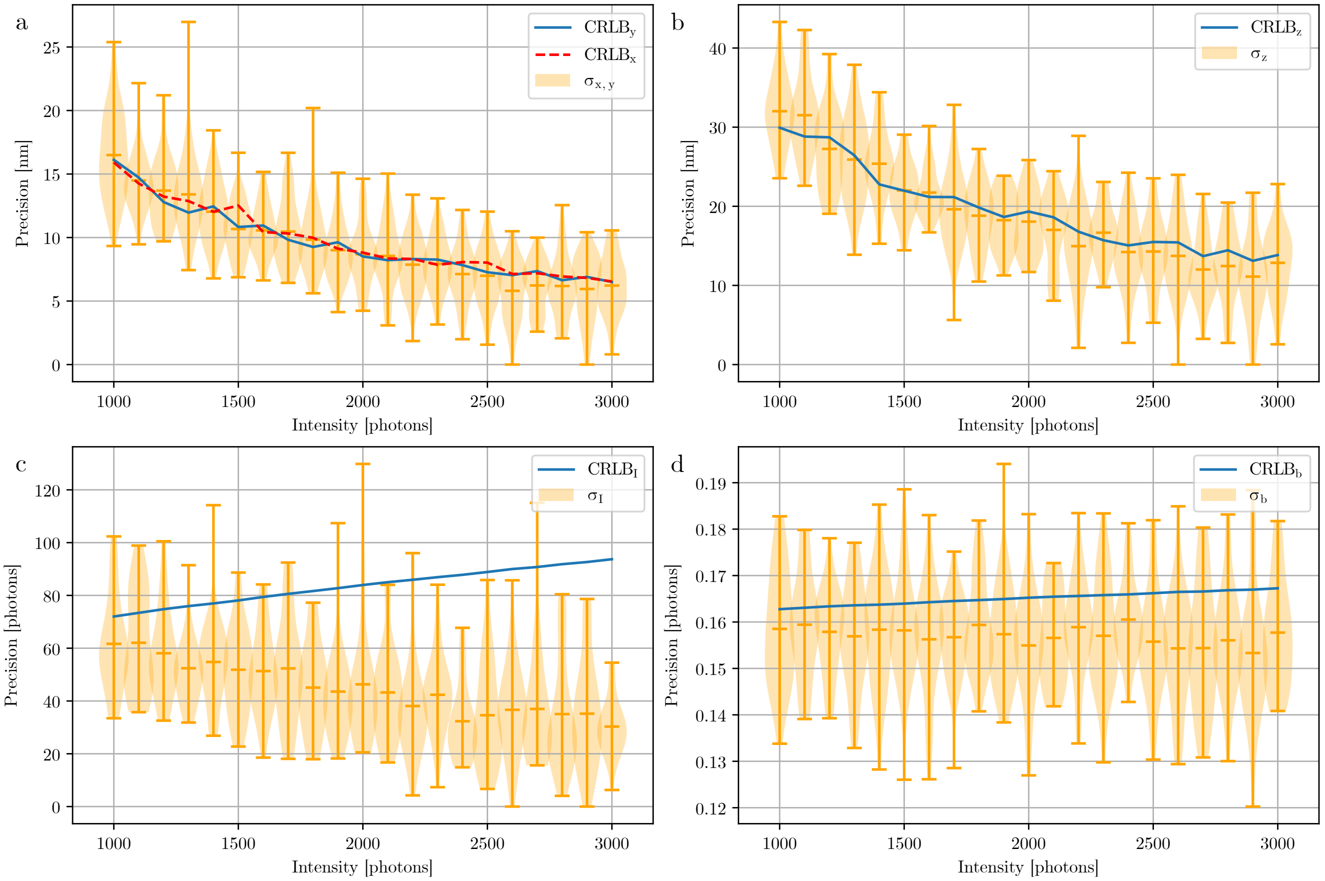

Supplementary Figure S10. Violinplots of the parameter estimates over intensity using a tetrapod PSF, compared to the CRLB. **a)** Lateral precision violinplot, using an effective pixel size of 100 nm. **b)** Axial precision violinplot. **c)** Violinplot of the emitter intensity precision. **d)** Violinplot of the background intensity precision. Precisions were calculated from the MCMC chain of individual frames, assuming a Gaussian distribution. With a background of 20 photons, 50 frames were used per datapoint from 500 to 3000 emitter intensity photons. Emitters were placed in focal plane at the center of the ROI and given a sub-pixel shift drawn from $\mathscr{U}(-0.5, 0.5)$.

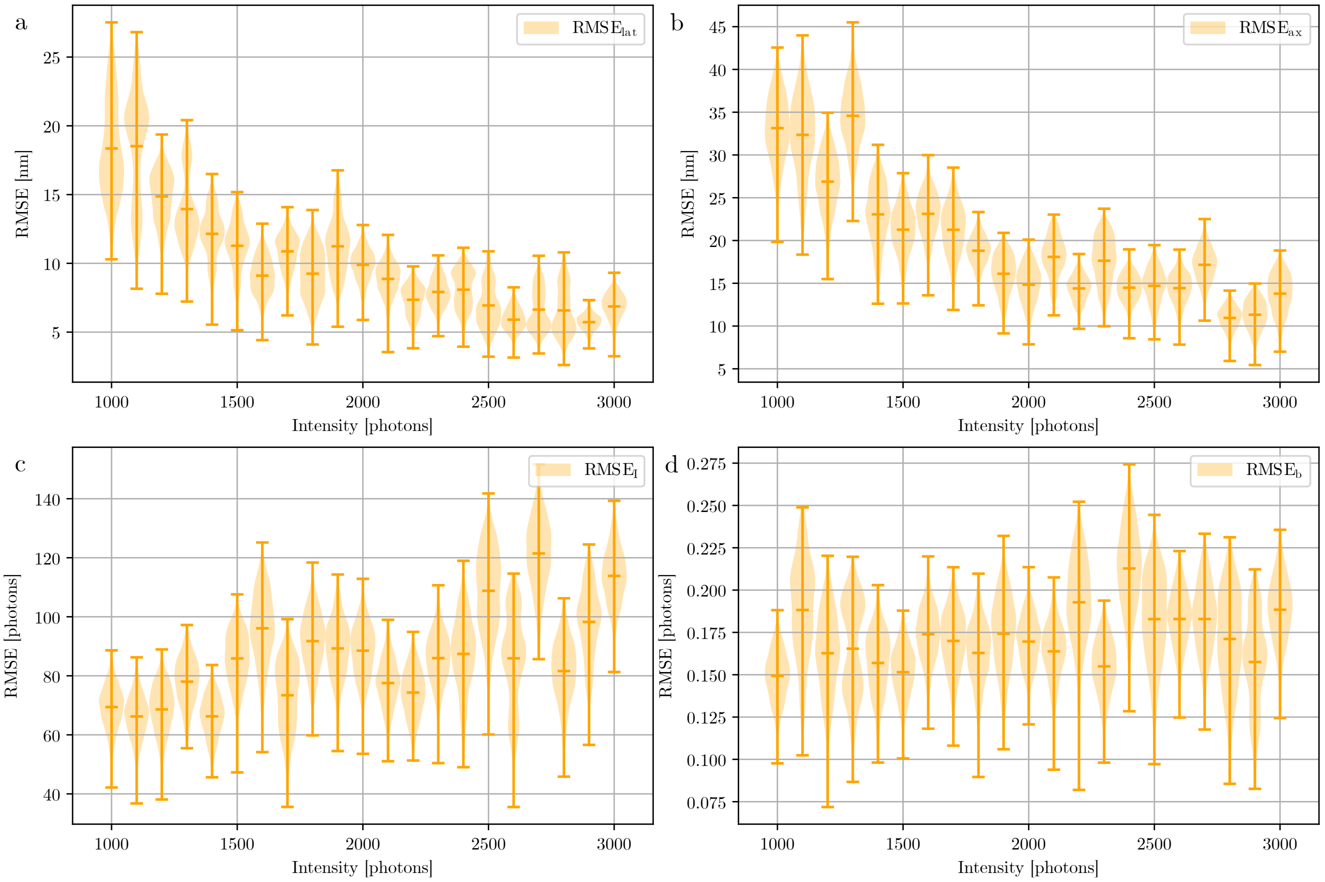

Supplementary Figure S11. Bootstrap resampled RMSE results for the parameter estimates using a tetrapod PSF, plotted over intensity. **a)** Violinplot of the lateral RMSE. **b)** Violinplot of the axial RMSE. **c)** Violinplot of the emitter intensity RMSE. **d)** Violinplot of the background intensity RMSE. Results were resampled using 50% of the available data and 1000 runs. With a background of 20 photons, 50 frames were used per datapoint from 500 to 3000 emitter intensity photons. Emitters were placed in focal plane at the center of the ROI and given a sub-pixel shift drawn from $\mathscr{U}(-0.5, 0.5)$.

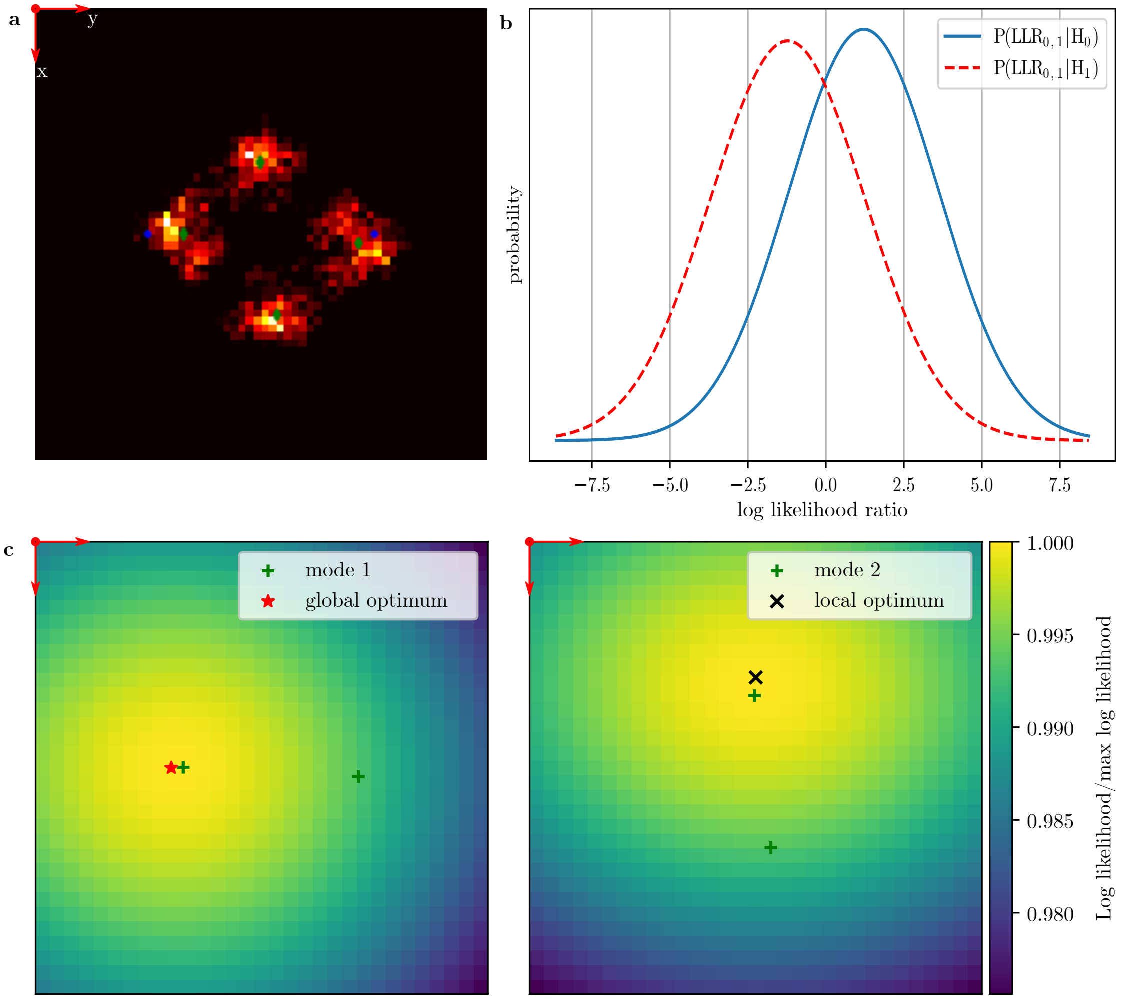

Supplementary Figure S12. Analysis of multimodality in astigmatic PSF 3D reconstructions for the two emitter problem. Ground truth positions are marked with blue dots, while localizations are marked with green crosses. **a)** Single frame reconstruction for the two emitter problem. **b)** Plots of the log likelihood ratios between the hypothesised modes of the reconstruction in a), using a Gaussian approximation. **c)** Left: fixing the rightmost localization, an emitter is shifted over the ROI, imaging the log likelihood and marking the maximum (red star). Right: the bottom emitter is fixed, and a local optimum is found in the black cross. This finds the pair of modes used in b), one on the ground truth and one perpendicular to it. Calculating the probability of error yields a 49.8% chance of selecting the wrong hypothesis. The expected value of either mode passes a chi-squared test on a 95% confidence interval.

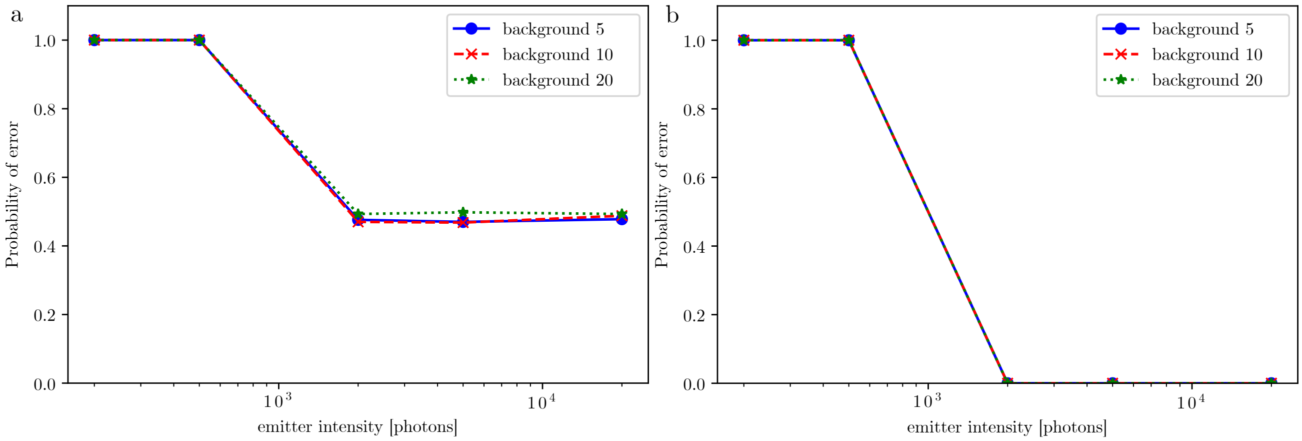

Supplementary Figure S13. Probability of error between modes for the two emitter problem using astigmatic and biplane imaging. Plotted for varying emitter and background intensities. **a)** Probability of error when selecting modes with astigmatic imaging. **b)** Probability of error when selecting modes using biplane imaging. Emitters were placed in focal plane, separated by 5/4 σ_{PSF} along the x-axis. All uniform priors except for emitter intensity, which used $\mathcal{N}(I_{sample}, 3.5\sqrt{I_{sample}})$. Probability of error was calculated using the same methods as for Supplementary Figure S12 described in the Supplementary Note on multimodality in two emitter astigmatic imaging. Reconstructions without alternate mode used a probability of error of 0, while reconstructions that could not separate emitters used a probability of error of 1.

Supplementary Table S1. Move proposal probabilities.

move type	probability		
	$RJMCMC_{burn}$	RJMCMC	MCMC
Single Emitter	$\frac{1}{5}$	$\frac{1}{4}$	$\frac{2}{5}$
Group Move	$\frac{1}{5}$	$\frac{1}{4}$	$\frac{2}{5}$
Background	$\frac{1}{5}$	$\frac{1}{4}$	$\frac{1}{5}$
Split	$\frac{1}{15}$	0	0
Merge	$\frac{1}{15}$	0	0
Generalized Split	$\frac{1}{15}$	$\frac{3}{32}$	0
Generalized Merge	$\frac{1}{15}$	$\frac{3}{32}$	0
Birth	$\frac{1}{15}$	$\frac{1}{32}$	0
Death	$\frac{1}{15}$	$\frac{1}{32}$	0

Supplementary Table S2. RJMCMC hyperparameters for astigmatic PSF, single emitter convergence test

	RJMCMC	MCMC	units
σ_x	0.05	0.05	[pixels]
σ_y	0.05	0.05	[pixels]
σ_z	0.08	0.07	[μm]
σ_I	18	15	[photons]
σ_b	1	1	[photons]
σ_{PSF}	1.3	1.3	[pixels]

Supplementary Table S3. RJMCMC hyperparameters for tetrapod PSF, single emitter convergence test

	RJMCMC	MCMC	units
σ_x	0.1	0.1	[pixels]
σ_y	0.1	0.1	[pixels]
σ_z	0.08	0.08	[μm]
σ_I	30	30	[photons]
σ_b	1	1	[photons]
σ_{PSF}	2.1	2.1	[pixels]

Supplementary Table S4. RJMCMC hyperparameters for astigmatic PSF, single emitter precision test

	RJMCMC	MCMC	units
σ_x	0.08	0.08	[pixels]
σ_y	0.08	0.08	[pixels]
σ_z	0.08	0.08	[μm]
σ_I	30	30	[photons]
σ_b	1	1	[photons]
σ_{PSF}	1.3	1.3	[pixels]

Supplementary Note

3D Reversible Jump Markov Chain Monte Carlo localization algorithm

To both estimate the model and find the parameters, Reversible Jump Markov Chain Monte Carlo (RJMCMC) is employed. The RJMCMC algorithm makes jumps in model space, making it possible to recover a distribution of possible model and parameter pairings. For 3D localization, the model only varies in number of emitters active per frame, in which we try to estimate the parameters of the emitters, that being the x, y, and z positions as well as the intensity I, and the background photons present in the frame, b. With the use of smart priors, 3D RJMCMC localization can retrieve the correct model and localize the emitters.

Localization in 3D using RJMCMC is done as follows:

1. An emitter is initialized randomly;
2. RJMCMC is run with the burn-in move probabilities;
3. The move probabilities are adjusted for the fraction of the chain that follows the burn-in;
4. From the post burn-in RJMCMC chain, the MAP model is selected from the distribution to be used with MCMC;
5. MCMC is initialized using the last element from the RJMCMC chain in which the model corresponds to the MAP model;
6. MCMC is run with the parameter space move list;
7. K-means clustering is used to perform label switching and retrieve the localizations from the chains, setting the cluster count to the MAP model.

The RJMCMC and MCMC runs undergo the following steps internally:

1. A move is randomly selected from the move list;
2. The move is executed on each frame, generating new parameters and models (if the move is not valid for the frame, the parameters stay fixed);
3. The acceptance rate is calculated and the jump is either accepted or rejected, updating the chain;
4. Steps 1 to 3 are repeated until the desired chain length is achieved.

Priors

The algorithm takes in priors for the emitter position, emitter intensity, background intensity, and the number of emitters present. Though the prior for number of emitters can be set as a Poisson distribution if the user has knowledge of the labelling density, we keep the prior $P(k)$ uniform as the local density when evaluating small regions of interest (ROIs) can differ greatly:

$$P(k) \sim \mathscr{U}(0, k_{\max}) \tag{1}$$

with $k_{\max}$ the user defined maximum number of emitters expected in the ROI.

For the emitter position, the priors are set to be uniform to avoid bias:

$$P(\theta_{x,i}|k) \sim \mathscr{U}(-w_{\text{border}}, x_{\text{ROI}} + w_{\text{border}}) \tag{2}$$

$$P(\theta_{y,i}|k) \sim \mathscr{U}(-w_{\text{border}}, y_{\text{ROI}} + w_{\text{border}}) \tag{3}$$

$$P(\theta_{z,i}|k) \sim \mathscr{U}(z_{\min}, z_{\max}) \tag{4}$$

with $\theta_{x,i}$, $\theta_{y,i}$, $\theta_{z,i}$ the 3D position of the ith found emitter, x_{ROI}, y_{ROI} the x- and y-dimensions of the ROI, w_{border} some additional pixels outside the ROI to account for influence of external emitters, and $z_{\min}$, $z_{\max}$ the admissible range of z-positions.

The prior on background intensity is kept as a simple uniform distribution:

$$P(\theta_b) \sim \mathscr{U}(b_{\min}, b_{\max}) \tag{5}$$

with $b_{\min}$, $b_{\max}$ the user defined minimum and maximum expected background values for the experiment.

Finally, the prior for the intensity should be either determined experimentally by kernel density fitting SMLM results, or directly defined by the user given sufficient knowledge of the emission response. For the testing on synthetic data, often (see Fazel et al.[1]) a Gaussian prior around the expected emitter intensity is used, with an additional flat shoulder at lower intensities to facilitate model jumps if necessary, such as:

$$P(\theta_{I,i}|k) = \begin{cases} \frac{1}{150\sqrt{2\pi}} \exp\left[-\frac{1}{2}\left(\frac{\theta_I - 2000}{150}\right)^2\right], & \text{if } 1600 < \theta_I < 2500 \\ 6 \cdot 10^{-5}, & \text{if } 500 < \theta_I < 1600 \\ 6 \cdot 10^{-5} \frac{\theta_I}{500}, & \text{if } 1 < \theta_I < 500 \\ 0, & \text{otherwise} \end{cases} \quad (6)$$

The prior on intensity is assumed to be unchanging over depth.

Moves

In RJMCMC, the design of the moves is crucial for exploring the model space. The moves used in 3D RJMCMC localization are:

- A single emitter move, varying the 3D position and intensity of a single emitter;
- A group move, varying the 3D position and intensity of a cluster of emitters;
- A background move, changing the offset background estimate;
- A split move, splitting one emitter into two;
- A merge move, merging two emitters into one;
- A generalized split move, splitting a cluster of N emitters into $N+1$;
- A generalized merge move, merging a cluster of N emitters into $N-1$;
- A birth move, spawning an emitter where the difference between measured and expected intensity is high;
- A death move, removing an emitter at random.

The single emitter, group, and background move work in parameter space while leaving the model space identical. The rest of the moves come in pairs to retain detailed balance and change both the model and the parameters of the estimate. Supplementary Figure S1 shows schematically how each move changes the parameters or model.

Move proposal At every iteration of the algorithm, a move is selected according to fixed probabilities defined by the user. Move probabilities can be set separately for the MCMC portion, as well as both the burn-in phase and the post burn-in phase of the RJMCMC portion.
An appropriate setting of the move probabilities results in sufficient mixing between chains. As a result of chain mixing, the algorithm makes jumps between multiple solution modes, which allows exploration of the full posterior density. Specifically for our problem, this allows us to explore ambiguous localization solutions due to PSF degeneracy.
To facilitate sufficient mixing, the following procedure was used to determine the move probabilities in Supplementary Table S1. To choose the probabilities related to 2D moves, the parameters from Fazel et al.[1] were initially chosen. For the 3D probabilities, the starting point was based on our presumed depth of view. Given the increased complexity when moving from 2D RJMCMC to 3D RJMCMC, we identified that these parameters needed to be tuned. As such, these parameters were tuned until sufficient mixing between the chains was observed. For testing on synthetic data, the move probabilities in Supplementary Table S1 were found to work well.

Single emitter move

The single emitter, group, and background move moves will act on the parameter space only and their acceptance rates can be determined from the Metropolis-Hastings algorithm for the MCMC case.

Metropolis-Hastings acceptance rate for parameter space jumps The probability of accepting parameter space jumps is given as:

$$\alpha = \min\left[1, \frac{P(\theta', k, D)}{P(\theta, k, D)}\left|\frac{\partial(\theta')}{\partial(\theta, u)}\right|\right] \tag{7}$$

with α the acceptance rate, using the joint probability $P(\theta,k,D) = P(D|\theta,k)P(\theta|k)P(k)$ to calculate the posterior ratio (ignoring evidence term $P(D)$ as it gets cancelled in the division) and the final fraction as the determinant of the Jacobian of the function $\theta'(\theta,u)$ that generates the new parameters. Here, $\theta = \begin{bmatrix}\theta_{x,0} & \theta_{y,0} & \theta_{z,0} & \theta_{I,0} & \dots & \theta_{x,i} & \theta_{y,i} & \theta_{z,i} & \theta_{I,i} & \theta_b\end{bmatrix}$ is the parameter vector, containing the positions and intensities of the i emitters used in the current model along with the background parameter θ_b. Using a random walk sampler for the parameter space moves and cancelling the model prior, the acceptance rate reduces to:

$$\alpha = \min\left[1, \frac{P(\theta', k, D)}{P(\theta, k, D)}\right] = \min\left[1, \frac{P(D|\theta', k)P(\theta'|k)}{P(D|\theta, k)P(\theta|k)}\right] \tag{8}$$

The single emitter move randomly selects one of the i current emitters and generates a new estimate out of its previous parameters as follows:

$$\theta'_{x,j} = \theta_{x,j} + u_x \tag{9}$$
$$\theta'_{y,j} = \theta_{y,j} + u_y \tag{10}$$
$$\theta'_{z,j} = \theta_{z,j} + u_z \tag{11}$$
$$\theta'_{I,j} = \theta_{I,j} + u_I \tag{12}$$

with u drawn from the following normal distributions:

$$P(u_x) \sim \mathcal{N}(0, \sigma_x) \tag{13}$$
$$P(u_y) \sim \mathcal{N}(0, \sigma_y) \tag{14}$$
$$P(u_z) \sim \mathcal{N}(0, \sigma_z) \tag{15}$$
$$P(u_I) \sim \mathcal{N}(0, \sigma_I) \tag{16}$$

and σ_x, σ_y, σ_z, σ_I some user determined hyperparameters to vary the jump size. The posterior ratio in Equation 8 can now be simplified to:

$$\frac{P(D|\theta', k)P(\theta'|k)}{P(D|\theta, k)P(\theta|k)} = \left[\prod_{k_\mathrm{p}} \exp\left(\mu_{k_\mathrm{p}} - \mu'_{k_\mathrm{p}}\right)\left(\frac{\mu'_{k_\mathrm{p}}}{\mu_{k_\mathrm{p}}}\right)^{n_{k_\mathrm{p}}}\left(\prod_{k}^{k_\mathrm{max}} \frac{P(\theta'_{I,k})}{P(\theta_{I,k})}\right)\right] \tag{17}$$

Assuming the estimated parameters stay within the bounds of the priors, the uniform position priors cancel out and only the likelihood ratio and intensity prior ratio are left.

Group move

The group move first finds a cluster of nearby emitters, and then executes a single emitter move on each of them. It randomly picks one emitter, then searches for emitters within a $2\sigma_{\mathrm{PSF}}$ sphere around it to form a cluster. This radius can be treated as a hyperparameter and tuned, though for Gaussian and astigmatic PSFs it works well when set to the PSF width in focal plane. More complex 3D PSFs may require a fairly large radius for clustering. After finding an eligible cluster and executing the moves, the acceptance rate can be calculated using the same equations as in the single move, namely as in Equations 8 and 17. If a cluster cannot be found, the move fails.

Background move

The background move is not coupled to any emitter. It updates a flat offset background intensity with a random walk sampler as follows:

$$\theta'_b = \theta_b + u_b \tag{18}$$
$$P(u_b) \sim \mathcal{N}(0, \sigma_b) \tag{19}$$

with σ_b another hyperparameter for tuning the background jump size. The acceptance rate from Equation 17 can now be reduced even further, assuming the background jump stays within the bounds of its uniform prior:

$$\frac{P(D|\theta',k)P(\theta'|k)}{P(D|\theta,k)P(\theta|k)} = \left[\prod_{k_\mathrm{p}} \exp\left(\mu_{k_\mathrm{p}} - \mu'_{k_\mathrm{p}}\right)\left(\frac{\mu'_{k_\mathrm{p}}}{\mu_{k_\mathrm{p}}}\right)^{n_{k_\mathrm{p}}}\right] \tag{20}$$

which is just the likelihood ratio of the parameters before and after the jump.

Split and Merge pair

The (generalized) split, merge, birth and death moves come in pairs and may act on both the parameter and the model space. Their acceptance rates can be determined from the general acceptance rate used in RJMCMC.

Metropolis-Hastings acceptance rate for model space jumps The probability of accepting model space jumps in the general case is given as:

$$\alpha = \min\left\{1, \frac{P(\theta',k',D)r_\mathrm{m}(\theta')}{P(\theta,k,D)r_\mathrm{m}(\theta)q(u)}\left|\frac{\partial(\theta')}{\partial(\theta,u)}\right|\right\} \tag{21}$$

with $r_\mathrm{m}(\theta)$ the probability of choosing the move type (selecting either split or merge) and $q(u)$ the probability density function of the draws from u used to generate the split weights, means, and standard deviations. The split and merge move turn one emitter into two and vice versa. By using this move pair, the algorithm has a move that allows it to distinguish emitters that are in close proximity to one another. Split and merge will uphold the following constraints:

$$\theta_{I,j^*} = \theta_{I,j_1} + \theta_{I,j_2} \tag{22}$$
$$\theta_{I,j^*}\theta_{x,j^*} = \theta_{I,j_1}\theta_{x,j_1} + \theta_{I,j_2}\theta_{x,j_2} \tag{23}$$
$$\theta_{I,j^*}\theta_{y,j^*} = \theta_{I,j_1}\theta_{y,j_1} + \theta_{I,j_2}\theta_{y,j_2} \tag{24}$$
$$\theta_{I,j^*}\theta_{z,j^*} = \theta_{I,j_1}\theta_{z,j_1} + \theta_{I,j_2}\theta_{z,j_2} \tag{25}$$
$$\tag{26}$$

Split For a split, θ' is generated from a randomly selected emitter with index j^* as follows:

$$u_1 \sim \mathscr{U}(0,1) \tag{27}$$
$$u_2 \sim \mathscr{N}(0,\sigma_\mathrm{PSF}) \tag{28}$$
$$u_3 \sim \mathscr{N}(0,\sigma_\mathrm{PSF}) \tag{29}$$
$$u_4 \sim \mathscr{N}(0,\sigma_\mathrm{PSF}) \tag{30}$$
$$\theta_{I,j_1} = \theta_{I,j^*}u_1 \tag{31}$$
$$\theta_{I,j_2} = \theta_{I,j^*}(1-u_1) \tag{32}$$
$$\theta_{x,j_1} = \theta_{x,j^*} + u_2 \tag{33}$$
$$\theta_{y,j_1} = \theta_{y,j^*} + u_3 \tag{34}$$
$$\theta_{z,j_1} = \theta_{z,j^*} + u_4 \tag{35}$$
$$\theta_{x,j^*}\theta_{I,j^*} = \theta_{x,j_1}\theta_{I,j_1} + \theta_{x,j_2}\theta_{I,j_2} \tag{36}$$
$$\theta_{x,j_2} = \theta_{x,j^*} - \frac{u_1u_2}{1-u_1} \tag{37}$$
$$\theta_{y,j^*}\theta_{I,j^*} = \theta_{y,j_1}\theta_{I,j_1} + \theta_{y,j_2}\theta_{I,j_2} \tag{38}$$
$$\theta_{y,j_2} = \theta_{y,j^*} - \frac{u_1u_3}{1-u_1} \tag{39}$$
$$\theta_{z,j^*}\theta_{I,j^*} = \theta_{z,j_1}\theta_{I,j_1} + \theta_{z,j_2}\theta_{I,j_2} \tag{40}$$
$$\theta_{z,j_2} = \theta_{z,j^*} - \frac{u_1u_4}{1-u_1} \tag{41}$$

with the two new emitters retaining the 3D center of mass and total intensity of the emitter they split from. Indices j_1 and j_2 are used to indicate the emitters resulting from a split, or those used in a merge, whereas index j^* is used for the result of a merge or the target of a split.

The ratio of selecting the move types then becomes:

$$\frac{r_{\mathrm{m}}(\theta')}{r_{\mathrm{m}}(\theta)} = \frac{P_{\mathrm{split}}}{P_{\mathrm{merge}}} \tag{42}$$

and $q(u)$ is:

$$q(u) = P(u_1)P(u_2)P(u_3)P(u_4) \tag{43}$$

Knowing the split move only influences these parameters and leaves the other emitters and background untouched, the emitter to be split and the resulting emitters after the split can be moved to the back for both θ' and (θ, u) vectors. The resulting vectors then become:

$$\theta' = \begin{bmatrix} \dots & \theta_{I,j_1} & \theta_{x,j_1} & \theta_{y,j_1} & \theta_{z,j_1} & \theta_{I,j_2} & \theta_{x,j_2} & \theta_{y,j_2} & \theta_{z,j_2} \end{bmatrix} \tag{44}$$

$$(\theta, u) = \begin{bmatrix} \dots & \theta_{I,j^*} & u_1 & \theta_{x,j^*} & u_2 & \theta_{y,j^*} & u_3 & \theta_{z,j^*} & u_4 \end{bmatrix} \tag{45}$$

The Jacobian can then be constructed as:

$$\frac{\partial(\theta')}{\partial(\theta,u)} = \begin{bmatrix} I_{k-1} & \mathbf{0}_{k-1,1} & & & & \dots & & & \mathbf{0}_{k-1,1} \\ \mathbf{0}_{1,k-1} & u_1 & 0 & 0 & 0 & -\theta_{I,j^*} & 0 & 0 & 0 \\ & 0 & 0 & 1 & 1 & 0 & 0 & 0 & 0 \\ & 0 & 0 & 0 & 0 & 1 & 1 & 0 & 0 \\ & 0 & 0 & 0 & 0 & 0 & 0 & 1 & 1 \\ \vdots & 1-u_1 & 0 & 0 & 0 & -\theta_{I,j^*} & 0 & 0 & 0 \\ & 0 & \frac{-u_2}{(1-u_1)^2} & 1 & \frac{-u_1}{1-u_1} & 0 & 0 & 0 & 0 \\ & 0 & \frac{-u_3}{(1-u_1)^2} & 0 & 0 & 1 & \frac{-u_1}{1-u_1} & 0 & 0 \\ \mathbf{0}_{1,k-1} & 0 & \frac{-u_4}{(1-u_1)^2} & 0 & 0 & 0 & 0 & 1 & \frac{-u_1}{1-u_1} \end{bmatrix}$$

with identity matrix I_{k-1} of dimension $k-1$ for all the unchanged parameters and $\mathbf{0}_{k-1,1}$, $\mathbf{0}_{1,k-1}$ column and row vectors containing zeros. This yields the determinant:

$$\left| \frac{\partial(\theta')}{\partial(\theta,u)} \right| = \frac{\theta_{I,j^*}}{(1-u_1)^3} \tag{46}$$

Setting $P_{\mathrm{split}} = P_{\mathrm{merge}}$, the final result is then:

$$\alpha = \min\left\{1, A_{\mathrm{split}}\right\} \tag{47}$$

$$A_{\mathrm{split}} = \frac{P(\theta'|D) r_{\mathrm{m}}(\theta')}{P(\theta|D) r_{\mathrm{m}}(\theta) q(u)} \left| \frac{\partial(\theta')}{\partial(\theta,u)} \right| = \frac{P(\theta',k',D)}{P(\theta,k,D)} \frac{\theta_{I,j^*}}{P(u_1,u_2,u_3,u_4)(1-u_1)^3} \tag{48}$$

Merge For a merge move, an emitter is selected at random, after which it is randomly paired with another emitter within 2 σ_{PSF} of itself. If no emitter can be found within this distance, the move fails. The emitters combine their intensity and center of mass. The acceptance rate can be set to the inverse of that for the split move, finding u_1, u_2, u_3 and u_4 deterministically from Equations 31, 33, 34, and 35:

$$u_1 = \frac{\theta_{I,j_1}}{\theta_{I,j^*}} \tag{49}$$

$$u_2 = \theta_{x,j_1} - \theta_{x,j^*} \tag{50}$$

$$u_3 = \theta_{y,j_1} - \theta_{y,j^*} \tag{51}$$

$$u_4 = \theta_{z,j_1} - \theta_{z,j^*} \tag{52}$$

This gives the the acceptance rate:

$$\alpha = \min\left\{1, A_{\mathrm{merge}}\right\} \tag{53}$$

$$A_{\mathrm{merge}} = A_{\mathrm{split}}^{-1} = \frac{P(\theta,k,D)}{P(\theta',k',D)} \frac{P(u_1,u_2,u_3,u_4)(1-u_1)^3}{\theta_{I,j^*}} \tag{54}$$

with θ previously being the parameters before the split and now thus the parameters after the merge and vice versa for θ'.

Generalized Split and Merge

Whereas split and merge work on a single emitter, their generalized versions are helpful for very densely packed frames, allowing the splitting and merging of emitters within clusters of N emitters. Generalized split jumps from N to $N+1$ emitters, while generalized merge does the opposite. These moves use the same clustering as was used for the group move, picking one random emitter and forming an eligible cluster within $2\sigma_{\text{PSF}}$ of itself.

Generalized Split Now, instead of distributing the intensity of one emitter to two, the combined intensity of N emitters is distributed over $N+1$, again retaining the center of mass:

$$\sum_{j=1}^{N} \theta_{I,j} = \sum_{j=1}^{N+1} \theta'_{I,j} \tag{55}$$

$$\sum_{j=1}^{N} \theta_{I,j}\theta_{x,j} = \sum_{j=1}^{N+1} \theta'_{I,j}\theta'_{x,j} \tag{56}$$

$$\sum_{j=1}^{N} \theta_{I,j}\theta_{y,j} = \sum_{j=1}^{N+1} \theta'_{I,j}\theta'_{y,j} \tag{57}$$

$$\sum_{j=1}^{N} \theta_{I,j}\theta_{z,j} = \sum_{j=1}^{N+1} \theta'_{I,j}\theta'_{z,j} \tag{58}$$

Putting up some additional constraints to remove the same fraction of intensity for each emitter in the cluster and to let the new emitter be formed close to the center of mass of the cluster:

$$\theta'_{I,j} = (1-u_1)\theta_{I,j} \tag{59}$$

$$\theta'_{x,j} = \frac{\theta_{x,j} - u_1\left(\frac{1}{N}\sum_{j=1}^{N} \theta_{x,j} + u_2\right)}{1-u_1} \tag{60}$$

$$\theta'_{y,j} = \frac{\theta_{x,j} - u_1\left(\frac{1}{N}\sum_{j=1}^{N} \theta_{x,j} + u_3\right)}{1-u_1} \tag{61}$$

$$\theta'_{z,j} = \frac{\theta_{y,j} - u_1\left(\frac{1}{N}\sum_{j=1}^{N} \theta_{z,j} + u_4\right)}{1-u_1} \tag{62}$$

The new emitter is then generated as follows:

$$\theta'_{I,N+1} = u_1 \sum_{j=1}^{N} \theta_{I,j} \tag{63}$$

$$\theta'_{x,N+1} = \frac{1}{N}\sum_{j=1}^{N} \theta_{x,j} + u_2 \tag{64}$$

$$\theta'_{y,N+1} = \frac{1}{N}\sum_{j=1}^{N} \theta_{y,j} + u_3 \tag{65}$$

$$\theta'_{z,N+1} = \frac{1}{N}\sum_{j=1}^{N} \theta_{z,j} + u_4 \tag{66}$$

Applying the general acceptance rate from Equation 21, the result is similar to Equation 48 except the Jacobian now expands to:

$$\left|\frac{\partial(\theta')}{\partial(\theta,u)}\right| = \frac{\sum_{j=1}^{N} \theta_{I,j}}{(1-u_1)^{2N+1}} \tag{67}$$

The acceptance rate then becomes:

$$A_{\text{g-split}} = \frac{P(\theta',k',D)}{P(\theta,k,D)} \frac{\sum_{j=1}^{N} \theta_{I,j}}{P(u_1,u_2,u_3,u_4)((1-u_1)^{2N+1}} \tag{68}$$

Generalized Merge The generalized merge selects an emitter and then distributes its intensity evenly over the N emitters in the corresponding cluster. As with the merge, the randomly sampled parameters can now be found deterministically:

$$u_1 = \frac{\theta'_{I,N+1}}{\sum_{j=1}^{N} \theta_j} \tag{69}$$

$$u_2 = \theta'_{x,N+1} - \frac{1}{N}\sum_{j=1}^{N} \theta_{x,j} \tag{70}$$

$$u_3 = \theta'_{y,N+1} - \frac{1}{N}\sum_{j=1}^{N} \theta_{y,j} \tag{71}$$

$$u_4 = \theta'_{z,N+1} - \frac{1}{N}\sum_{j=1}^{N} \theta_{z,j} \tag{72}$$

The new parameters become:

$$\theta_{I,j} = \frac{\theta'_{I,j}}{1-u_1} \tag{73}$$

$$\theta_{x,j} = u_1\theta'_{x,N+1} + (1-u_1)\theta'_{x,j} \tag{74}$$

$$\theta_{y,j} = u_1\theta'_{x,N+1} + (1-u_1)\theta'_{y,j} \tag{75}$$

$$\theta_{z,j} = u_1\theta'_{x,N+1} + (1-u_1)\theta'_{z,j} \tag{76}$$

$$\tag{77}$$

Inverting the acceptance rate of the generalized split yields:

$$A_{\text{g-merge}} = \frac{P(\theta,k,D)}{P(\theta',k',D)} \frac{P(u_1,u_2,u_3,u_4)((1-u_1)^{2N+1}}{\sum_{j=1}^{N} \theta_{I,j}} \tag{78}$$

Birth and Death pair

The birth and death pair can increase and decrease the number of emitters independent of the positions of the currently found emitters. Birth will generate an emitter anywhere in the volume covered by the support of the position priors, using the residual of the frame and the expected image as the probability distribution for selecting the new lateral emitter position. Death randomly chooses any of the current emitters and removes it.

Birth The first task of the birth move is finding probable locations for yet unmodelled emitters. It calculates the expected image from the current model and parameters and subtracts this from the ROI. Any negative values are fixed to zero, and the values are normalized to give a per pixel probability of an undetected emitter:

$$P(k_{\text{new},k_\text{p}}) = \frac{n_{k_\text{p}} - \mu_{k_\text{p}}}{\sum_{k_\text{p}=1}^{N_\text{p}} n_{k_\text{p}} - \mu_{k_\text{p}}}, \qquad \{k_\text{p} : n_{k_\text{p}} - \mu_{k_\text{p}} \geq 0\} \tag{79}$$

$$\tag{80}$$

with $P(k_{\text{new},k_\text{p}})$ the pixel probability of a yet undiscovered emitter residing in pixel p_k. After randomly selecting a pixel, a subpixel position is generated from a uniform distribution and the z-position is uniformly sampled from the entire depth range. The intensity of the new emitter is sampled directly from the intensity prior distribution.

This sampling of the z-position may result in a lot of ineffective proposals, however there are little effective alternatives to effectively finding the z-position that do not also impact calculation times or convergence. Some tests have been run where the residual image was filtered with the 3D PSF to return a probability distribution for pairs of x-, y- and z-position. It unfortunately resulted in some chains getting stuck, while only marginally improving convergence speed overall.

Fixing the birth and death selection probabilities to be equal, just as for the (generalized) split and merge moves, yields the acceptance rate:

$$A_{\text{birth}} = \frac{P(\theta',k',D)}{P(\theta,k,D)} \frac{z_{\max} - z_{\min}}{P(k^*_{\text{new},k_\text{p}})} \tag{81}$$

with $P(k^*_{\text{new},k_\text{p}})$ the probability of sampling from pixel k^*_p and z_max, z_min the depth range from which the z-position is sampled. Note that since birth and death work independently from the current emitters, the determinant of the Jacobian $\partial\theta'/\partial(\theta,u)$ is 1.

Death Death is the most straightforward move of them all, simply removing one emitter from the model at random. This move helps remove unnecessary emitters that may have a low intensity or contribute little to a cluster of emitters. It also helps remove emitters spawned by birth that do not necessarily fit the data, but went through despite a low acceptance rate. The acceptance rate for death is:

$$A_\text{death} = \frac{P(\theta',k',D)}{P(\theta,k,D)} = \frac{P(\theta'|D)}{P(\theta|D)} \tag{82}$$

where the second simplification holds as long as the model prior is kept uniform.

Single emitter localization convergence for an astigmatic PSF

To verify if the algorithm works correctly, it is first tested on frames where only a single emitter is active. Synthetic data is generated for an emitter in focal plane of a 20 by 20 pixel ROI, assuming an effective pixel size of 100 nm. Simulated emitters are placed in the center of the ROI, generating 50 frames, each with a random subpixel shift in x and y drawn from a uniform distribution and intensities drawn from a normal distribution:

$$\theta_x \sim \mathscr{U}(10-.5, 10+.5) \tag{83}$$
$$\theta_y \sim \mathscr{U}(10-.5, 10+.5) \tag{84}$$
$$\theta_I \sim \mathscr{N}(I_{\text{sim}}, 150) \tag{85}$$

with the position in pixels, and I_{sim} the mean intensity for the synthetic dataset. The background photon count is fixed at 20 photons. The algorithm is tested on data with $I_{\text{sim}} = 2000$ and $I_{\text{sim}} = 500$ to demonstrate convergence at varying SBR.

The hyperparameters used for localization are as follows. The RJMCMC portion ran for 20000 burn-in iterations, followed by another 10000 iterations from which the model is determined. MCMC is then run for another 5000 iterations, from which the final positions of the emitters are determined. The parameters for the random walk samplers within the RJMCMC and MCMC portions are detailed in Supplementary Table S2, while the move probabilities were taken from Supplementary Table S1. The depth was constrained to a range of $\begin{bmatrix}-1, & 1\end{bmatrix}$ μm.

The priors for position and background are uniform, with a custom prior used for intensity. The number of emitters was constrained to $k_{\max} = 6$. The algorithm was initialized randomly with $k_{\max}$ active emitters drawn from the priors, ensuring the initial model is incorrect and allowing for meaningful conclusions from the model time series and autocorrelation.

High SBR results

For $I_{\text{sim}} = 2000$, the prior on intensity is given as follows:

$$P(I) = \begin{cases} 0, & \text{if } I > 2500 \\ \frac{1}{150\sqrt{2\pi}} \exp\left\{-\frac{1}{2}\left(\frac{I-2000}{150}\right)^2\right\}/1.08, & \text{if } 1600 < I < 2500 \\ 6.6 \cdot 10^{-5}, & \text{if } 500 < I < 1600 \\ \left(\frac{I}{500}\right) \cdot 6.6 \cdot 10^{-5}, & \text{if } I > 500 \end{cases} \tag{86}$$

The prior is plotted in Supplementary Figure S2.

Model convergence To verify the model has converged, the time series of the models over all frames as well as the average autocorrelation of the model is studied. In Supplementary Figure S3, the model time series and autocorrelation are plotted. From the time series, we can see that from the initialization at $k_{\max} = 6$ emitters, the estimated model rapidly reduces to a two or one emitter model. There is notably little mixing in the model chain. This may be due to the simplicity of localizing a single emitter, the algorithm therefore having infinitesimal probabilities of jumping to models with 3 emitters or more. Note that after the burn-in fraction, the split and merge move are turned off and there is no more change in the model found. With split and merge disabled, and G-split and G-merge failing since no clusters can be made out of one emitter, the only moves capable of changing the model are birth and death. At this point, the RJMCMC seems to have converged strongly. Evaluating the average model chain autocorrelation, it is clear that after 2000 RJMCMC iterations, the autocorrelation has gone to zero. This gives an idea of the chain length required to estimate the model distribution under these conditions. Some multiple of 2000, say 6000 RJMCMC iterations, may already be sufficient for model convergence on this problem. From the histogram in Supplementary Figure S3 d) it is clear that on average, the estimates spend nearly all their time in a model with one active emitter.

Parameter convergence Again, we look to the autocorrelation of the parameters to assess convergence. In Supplementary Figure S3 b), the average autocorrelations of all parameters for their MCMC chains are plotted. Note that the MCMC chain was initalized from the last RJMCMC iteration that had the correct model, therefore the parameters can be expected to converge quickly and there is no need to discard the initial part of the chain. The autocorrelations of the lateral position and background decrease almost immediately to zero. Note that while the autocorrelation for axial position drops to zero in the same time, there are some slower transients visible in the plot. The same holds for the autocorrelation of intensity, which also takes significantly longer to reach zero. The acceptance rates for the moves are $\begin{bmatrix}0, & 0.47 \pm 0.02, & 0.28 \pm 0.01\end{bmatrix}$ for the group move, single emitter move, and background move, respectively. The group move cannot find a cluster and is therefore never executed, meanwhile the single emitter move has a good acceptance rate ensuring proper mixing. The background move has low acceptance, though it should still be enough for proper mixing.

Calculating the lateral and axial RMSE results in 0.05 pixels and 120 nm errors, respectively, giving us an accurate estimate of the emitter position.

It can be concluded that the initial position found from RJMCMC was sufficiently close to the optimum and the chosen hyperparameters and chain length were appropriate, though the sampling of intensity still shows correlation and σ_I may need to be tuned more.

Low SBR results

For $I_{\text{sim}} = 500$, the prior on intensity is given as follows:

$$P(I) = \begin{cases} 0, & \text{if } I > 950 \\ \frac{1}{150\sqrt{2\pi}} \exp\left\{ -\frac{1}{2}\left(\frac{I-500}{150}\right)^2 \right\}, & \text{if } I < 950 \end{cases} \tag{87}$$

The prior is plotted in Supplementary Figure S4. This dataset samples intensities from $\mathcal{N}(500, 150)$ photons, with a background of 20 photons.

Model convergence In Supplementary Figure S5 a) and c), the model time series and autocorrelation are plotted. Again, from the incorrect initalization the estimated model rapidly reduces to a two or one emitter model. There is less mixing and faster convergence compared to the simulations with $I_{\text{sim}} = 2000$. Looking at the average autocorrelation, it reaches zero with a lag of just 250. Again, this indicates that the 30000 RJMCMC iterations are more than sufficient for determining the model. The histogram in Supplementary Figure S5 d) again shows that the chain spends virtually all its time in a single emitter model.

Parameter convergence In Supplementary Figure S5 b), the average parameter autocorrelation for the 50 frames is shown. The autocorrelations of the position chains take longer to reach zero compared to the high SBR case. This time, the axial chain is actually slower than the intensity chain, and now even the lateral chain displays some slow transient behavior. The background converges first, followed by intensity and lateral position, and finally axial position. Under these conditions, convergence is limited by the sampling of the z-position instead of the intensity. The acceptance rates for the moves are $\begin{bmatrix}0, & 0.73 \pm 0.04, & 0.270 \pm 0.009\end{bmatrix}$. The single emitter move has a high acceptance rate and the corresponding random walk hyperparameters may be increased for better mixing and less correlation between samples.

Calculating the lateral and axial RMSE results in 2 pixels and 360 nm errors, respectively, which is a lot less accurate especially in lateral direction compared to the high SBR case. This is due to some of the outliers in the sampled intensity, for instance frames 18 and 45 having an intensity less than 100 photons (79 and 83, respectively). Not considering these two frames, intensities still range from 200 to 700 photons (covering about 85% of the interval), and the lateral and axial RMSE become 0.14 pixels and 340 nm each.

This test has shown that though the chain length is sufficient, tuning of the single emitter move may lead to faster convergence.

Finally, it can be concluded that the hyperparameters provided here generally result in convergence, though it is best practice to adjust them to the imaging conditions of your system, ideally making use of autocorrelation plots to identify sub-optimal behaviour.

Single emitter localization convergence for a tetrapod PSF

The same test is run for a tetrapod PSF, under similar conditions, simulating 50 frames of in-focus emitters in the center of the ROI with some subpixel shift, for varying SBR. The size of the ROI was set to 28 by 28 pixels, to account for the wider PSF. The same move probabilities were used, while adjusting the hyperparameters and PSF width, which was estimated from a Gaussian fit of the width in focal plane. The hyperparameters can be found in Supplementary Table S3.

High SBR results

For $I_{\text{sim}} = 2000$, using the intensity prior shown in Supplementary Figure S2, the results are as follows.

Model convergence Studying the autocorrelation in Supplementary Figure S6 a), it appears that model convergence is slightly slower with the tetrapod PSF. This may be related to use of a fixed clustering radius, after all the tetrapod PSF greatly changes in width over depth. There is a lot more mixing in the chain of Supplementary Figure S6 c) this time, with two or three emitter models existing up to around 3000 iterations into the run. Beyond 5000 iterations, the RJMCMC run seems to have converged strongly. Evaluating the average model chain autocorrelation suggests that a minimum of 2600 RJMCMC iterations is needed. Some multiple of 2600, say 7800 RJMCMC iterations, should be sufficient for model convergence on this problem. From the histogram in Supplementary Figure S6 d) it is clear that on average, the estimates spend nearly all their time in a model with one active emitter.

Parameter convergence Supplementary Figure S6 b) plots the average parameter autocorrelation for the 50 frames. The lateral, axial, and background autocorrelation converge rapidly. The intensity chain takes the longest to reach zero after a lag of 500 and still shows some more slow transient behavior. The acceptance rates for the moves are $\left[0, \quad 0.18 \pm 0.02, \quad 0.205 \pm 0.009\right]$ for the group move, single emitter move, and background move, respectively. The single emitter move has a low acceptance rate, possibly due to poor intensity sampling. The background move performs similarly to the runs on astigmatic data.

Calculating the lateral and axial RMSE results in 0.09 pixels and 20 nm errors.

It can be concluded that the chosen hyperparameters and chain length can converge, though the sampling of intensity still shows correlation and σ_I may need to be tuned more.

Low SBR results

For $I_{\text{sim}} = 500$, the prior in Supplementary Figure S4 was used. The results are as follows.

Model convergence In Supplementary Figure S7 a) and c), the model time series and autocorrelation are plotted. Again, from the incorrect initalization the estimated model rapidly reduces to a two or one emitter model. More mixing seems to be happening in the first 10000 iterations compared to the simulations with $I_{\text{sim}} = 2000$. The lower SBR gives more uncertainty when determining the model. The average model autocorrelation reaches zero at a lag of about 200, as expected from the increase in mixing. This again indicates that the 30000 iterations used were more than sufficient. The histogram in Supplementary Figure S7 d) shows that the chains generally stay in a single emitter model.

Parameter convergence In Supplementary Figure S7 b), the average parameter autocorrelation for the 50 frames is shown. This time, the lateral and axial position chains are slowest to converge, around a lag of 1500, while the background and intensity chains still reach zero in about 400 steps. Under these conditions, convergence is limited by the emitter position sampling instead of the intensity. The acceptance rates for the moves are $\left[0, \quad 0.55 \pm 0.08, \quad 0.198 \pm 0.009\right]$. Both the single emitter and background move show appropriate acceptance rates, the single emitter move even on the higher side. The high acceptance rates in combination with the strong correlation in parameter jumps suggests σ_x, σ_y, and σ_z may be increased to reduce sample correlation and get faster convergence.

The lateral and axial RMSE are in 1.6 pixels and 200 nm errors, respectively. Again, removing some outliers with intensities less than 200 returns precisions of 0.3 pixels and 70 nm, which is appropriate for these conditions.

Again, these tests have shown that extensive tuning of the single emitter move may lead to faster convergence in both scenarios.

Finally, the hyperparameters used for imaging with the tetrapod PSF will converge to an accurate estimate, though not as rapidly as astigmatic imaging with its respective hyperparameters.

Single emitter precision and accuracy for an astigmatic PSF

To further verify the accuracy and precision of the algorithm, another set of synthetic data was generated using the same PSFs from the convergence test. Again, single emitters with random subpixel shifts were simulated with a background of 20 photons and intensities ranging from 600 to 3000 photons, in steps of 100. 50 emitters were simulated at each intensity. Move probabilities were the same as in the convergence tests, while the hyperparameters can be found in Supplementary Table S4.

The intensity prior is now set to be uniform, fixing the range of the intensity prior from 100 to 4000 photons. This makes all the priors uniform, effectively only serving as loose constraints. It is now possible to compare our results to the Cramer-Rao lower bound (CRLB), as the algorithm is virtually unbiased. Supplementary Figure S8 plots the CRLB against the localization precision found directly on individual frames, calculated from the MCMC chain under a Gaussian assumption. This indicates that for the astigmatic PSF, the histograms from individual frames give an accurate representation of the achievable precision. Supplementary Figure S9 plots the resampled root mean squared error (RMSE) for the same results. The RMSE roughly matches the CRLB from Supplementary Figure S8, showing that the algorithm can find a precise and accurate estimate under these conditions.

Single emitter precision and accuracy for a tetrapod PSF

The same testing was done for a tetrapod PSF, using the same hyperparameters as in Supplementary Table S4 but with a PSF width of 2.1 pixels. At emitter intensities less than 1000 photons, some frames failed to converge, showcasing the difficulty of using a complex PSF at a low SBR. This may be due to the flat intensity prior yielding poor initializations and difficulty escaping local minima. The accuracy and precision are thus plotted over a range of 1000 to 3000 photons. Supplementary Figure S10 shows the CRLB and localization precision found as above. Note that the found precision for intensity breaks the CRLB, especially at higher intensity, despite the other parameter precisions tracking the CRLB well. The precision found when assuming a Gaussian distribution is thus not representative of the information present in the frame, at least not for the emitter intensity. Supplementary Figure S11 again shows the resampled RMSE for the same data. This time, all estimates behave as expected, broadly tracking the CRLB.

Multimodality in two emitter imaging

During testing of two emitter separability, under some conditions four peaks were found in the reconstruction of the emitter position. This occurred despite the chains in those situations converging to the correct MAP number of emitters. This phenomenon was observed at smaller distances between emitters, using the astigmatic PSF under relative angles of 0 and 90 degrees with respect to the x-axis, and for the tetrapod PSF under angles of 45 degrees. To understand if this was due to pseudoconvergence or if this was representative of the underlying posterior, a multimodal frame was analyzed, shown in Supplementary Figure S12. K-means clustering was used to find four potential localizations, shown together with the ground truth in Supplementary Figure S12 a). Then, by picking fixing one of these emitters and moving the other around the ROI at the same depth and intensity, the log likelihood was mapped, shown in Supplementary Figure S12 c). Fixing the rightmost emitter finds the global optimum to be a pair of emitters near the ground truth, while fixing the bottom emitter finds an alternate mode of the solution, a pair perpendicular to the ground truth. Note that this local optimum has virtually the same likelihood. To investigate if either mode could be representative of the data, a χ-squared test was done on a 95% confidence interval. The χ^2 value is calculated as follows:

$$\chi^2 = \sum_{i=1}^{N_\mathrm{p}} \frac{(D_i - \mu_i)^2}{\mu_i} \tag{88}$$

With N_p the number of pixels, D_i the measurement of the ith pixel, and μ_i the expected value. As the frames are subject to Poisson noise, the χ^2 threshold for a 95% confidence interval can be found with:

$$\chi^2_\mathrm{threshold} = N_\mathrm{p} + 1.96\sqrt{2N_\mathrm{p} + \sum_{i=1}^{N_\mathrm{p}} \frac{1}{\mu_i}} \tag{89}$$

Applying it to this frame results in $\chi^2_\mathrm{threshold} = 456$, with the true and alternate modes returning a χ^2 of 407 and 408, respectively. Both modes thus are well within the 95% confidence interval and are representative of the frame.

To test whether it is possible to select the true mode from these hypotheses, the log likelihood ratios of the hypotheses are used to calculate the probability of error. This log likelihood ratio can be approximated by a Gaussian distribution:

$$LLR_{q,r} = \ln\left(\frac{P(D|H_q)}{P(D|H_r)}\right) \tag{90}$$

$$\mu_{q,r,s} = \sum_{i}^{N_\mathrm{p}} \mu_i^s \ln\left(\frac{\mu_i^q}{\mu_i^r}\right) - \mu_i^q + \mu_i^r \tag{91}$$

$$\sigma_{q,r,s} = \sum_{i}^{N_\mathrm{p}} \mu_i^s \ln\left(\frac{\mu_i^q}{\mu_i^r}\right)^2 \tag{92}$$

with $LLR_{q,r}$ the log likelihood ratio of hypothesis q over hypothesis r, $\mu_{q,r,s}$ the mean of $LLR_{q,r}$ assuming hypothesis s is true, and $\sigma_{q,r,s}$ the corresponding width. For the two hypotheses H_0 and H_1, $P(LLR_{0,1}|H_0)$ and $P(LLR_{0,1}|H_1)$ can now be calculated, as shown in Supplementary Figure S12 b). To retrieve the probability of error, we now use the probability of making a correct selection:

$$P_\mathrm{c} = P(LLR_{0,1} > 0|H_0) - P(LLR_{0,1} < 0|H_1) \tag{93}$$

$$P_\mathrm{e} = (1 - P_\mathrm{c})/2 \tag{94}$$

with P_c the probability of being correct and P_e the probability of error. Applying this to the two modes from Supplementary Figure S12 results in a probability of error of 0.498. It is therefore not possible to select the right mode solely given the data.

Testing the multimodal reconstructions found under different angles for the astigmatic and tetrapod PSF yielded similar results, where both pairs of emitter locations pass the chi-squared test and have greater than 49.5% probability of error.

In conclusion, while both modes are representative of the data, there is no distinction to be made based on their likelihood. This indicates that pseudoconvergence is not the case and the posterior distribution in this scenario is multimodal. As both PSFs show this multimodality under different conditions, this indicates PSF degeneracy.

References

1. Fazel, M. *et al.* Bayesian multiple emitter fitting using reversible jump markov chain monte carlo. *Sci. reports* **9**, 1–10 (2019).
